# Mental health measures among adolescents in 12 low‐ and middle‐income countries: Measurement invariance and cross‐sectional analyses of Disrupting Harm survey data

**DOI:** 10.1002/jcv2.70087

**Published:** 2025-12-13

**Authors:** Ariadna Albajara Sáenz, Sebastian Kurten, Jennifer Saxton, Daniel Kardefelt‐Winther, Tamsin Ford, Amy Orben, Simon R. White

**Affiliations:** ^1^ Department of Psychiatry University of Cambridge Cambridge UK; ^2^ MRC Cognition and Brain Sciences Unit University of Cambridge Cambridge UK; ^3^ Department of Interdisciplinary Social Science Utrecht University Utrecht The Netherlands; ^4^ UNICEF Innocenti ‐ Global Office of Research and Foresight Florence Italy; ^5^ MRC Biostatistics Unit University of Cambridge Cambridge UK

**Keywords:** adolescents, low‐ and middle‐income countries, measurement invariance, mental health

## Abstract

**Background:**

Nationally representative mental health data in adolescents from low‐ and middle‐income countries (LMICs) are scarce. This study aimed to examine mental health and wellbeing indicators amongst adolescents in 12 LMICs across Eastern and Southern Africa and Southeast Asia.

**Methods:**

We conducted a secondary analysis of data involving 12,169 internet‐using adolescents, aged 12–17 years, from Ethiopia, Kenya, Mozambique, Namibia, Tanzania, Uganda, Cambodia, Indonesia, Malaysia, Philippines, Thailand, and Vietnam. We explored cross‐country measurement invariance of the multi‐item mental health scales, computed country‐level estimates for life satisfaction, psychological wellbeing, anxiety, depression, loneliness, self‐harm and suicidal ideation/attempts, and examined socio‐demographic variations (across age, gender and food insecurity).

**Results:**

Measurement invariance was not established, limiting cross‐country comparisons. No consistent patterns of mental health estimates emerged across countries. The greatest variation was observed for loneliness (ranging from 18.3% in Vietnam to 71.3% in the Philippines) and for suicidal ideation/attempts (ranging from 7% in Vietnam to 52.7% in Uganda). Gender and age disparities were present, but their magnitude and direction varied by country. The experience of food insecurity was the most consistent correlate of mental health outcomes, with significant associations with life satisfaction and psychological wellbeing in seven countries each, with anxiety in six, and with depression and loneliness in four.

**Conclusion:**

Overall, our findings provide key insights into the validity and use of a range of mental health measures among adolescents across 12 LMICs, as well as valuable within‐country observations that may help inform and tailor interventions, policies and future research on adolescent mental health to local contexts.

## Key points

**What's known?**

- Nationally representative mental health data on adolescents from low‐ and middle‐income countries are scarce.
- Understanding variations by gender, age, and food insecurity is essential to identify particularly vulnerable groups.

**What's new?**

- Measurement invariance was not supported for the multi‐item scales, limiting between‐country comparisons.
- No consistent patterns of mental health estimates emerged across countries; however, food insecurity was the most consistent correlate of mental health outcomes.

**What's relevant?**

- Our findings offer valuable within‐country insights into measurement of adolescent mental health across 12 LMICs, with respect to the validity and use of a range of measures that can help tailor research on adolescent mental health to local contexts.

## INTRODUCTION

Mental health disorders constitute the leading cause of morbidity among young people aged 10–24 years, with prevalence rates increasing over the past 30 years globally (Gore et al., 2011; Piao et al., 2022). In 2019, more than 1 in 10 individuals aged 10 to 19 reported at least one mental health disorder globally (Kieling et al., 2024). Poor mental health in adolescence often persists into adulthood and is associated with long‐term negative educational, occupational, financial, and social outcomes for the individual, and considerable societal economic costs (Asselmann et al., 2018; Copeland et al., 2021; Mulraney et al., 2021; UNICEF, 2021). Mental health and well‐being are now global development priorities within the United Nations' 2030 Agenda for Sustainable Development (United Nations, 2015).

Most available evidence on adolescent mental health comes from high‐income countries (HICs; Erskine et al., 2017). Nationally representative data from low‐and middle‐income countries (LMICs) are still scarce, due to limited routine data collection and research (Erskine et al., 2017; WHO, 2021a, 2022). Stigma and suicide criminalisation have been associated with underreporting of mental health difficulties (Seeman et al., 2016; WHO, 2022, 2023). Insufficient funding, unsupportive government policies, limited resources and the publication bias favouring HICs challenge research in LMICs (Abrahams & Nothling, 2024; Atilola, 2015; Hayes et al., 2023; Patel & Sumathipala, 2001; WHO, 2021a; Woelbert et al., 2020; Woelbert et al., 2021). The current extent of mental health need in adolescents in LMICs is less known, hindering the ability to inform appropriate resource allocation, service planning and support policies that address their needs, already limited in LMICs (Erskine et al., 2017; WHO, 2021a).

Age‐and sex‐related trends have been reported across a range of mental health measures in adolescents. During adolescence, mental health difficulties, particularly mood and anxiety disorders, increase, and subjective wellbeing and life satisfaction decrease, especially in girls (Campbell et al., 2021; Chen et al., 2019; Daly, 2022; Kieling et al., 2024; Newlove‐Delgado et al., 2023; Van Droogenbroeck et al., 2018; Yoon et al., 2023). Cross‐country differences in age trajectories and in the size and direction of the gender gap have also been reported (Campbell et al., 2021; Scott et al., 2018). Mental health data disaggregated by sex and age is under‐reported in LMICs (WHO, 2021a). However, such disaggregation is necessary to identify particularly vulnerable groups. Within both HICs and LMICs, lower socio‐economic status is associated with worse mental health (Henking et al., 2023). Food insecurity is common in LMICs, particularly in Africa and Asia (FAO et al., 2023; UNICEF, 2024). However, its association with mental health outcomes has been less studied among adolescents in LMICs.

The United Nations has declared a global imperative to improve mental health data collection and management globally (UNICEF, 2021; WHO, 2021a). Cross‐national mental health research poses methodological challenges including language barriers, sampling bias, and ethical issues, as well as variation in the conceptualisation of subjective constructs and response styles across different cultural groups (Atilola, 2015; Beins, 2011; Canino et al., 1997; Cummins & Lau, 2011; WHO, 2022). Assessing measurement invariance is necessary to establish the psychometric equivalence of a mental health measure across countries (Putnick & Bornstein, 2016). There has been limited testing for measurement invariance across countries of child and adolescent mental health scales, especially in LMICs, with evidence of cross‐cultural validity for only a few scales (Casas & González‐Carrasco, 2021; Stevanovic et al., 2017).

Using data from the Disrupting Harm project, this study aimed to examine mental health and wellbeing indicators among internet‐using adolescents aged 12–17 years in 12 LMICs across Africa and Asia. The objectives of this study were: (1) investigate cross‐country measurement invariance of the scales used, (2) provide country‐level mental health and wellbeing estimates, and (3) explore socio‐demographic variations by age, gender and food insecurity, providing a better understanding of the cross‐country performance and validity of the measures used.

## MATERIALS AND METHODS

### Study design and data source

This study analysed data from the Disrupting Harm project, implemented by ECPAT International (End Child Prostitution, Child Pornography And Trafficking of Children for Sexual Purposes), INTERPOL (The International Criminal Police Organisation), and UNICEF Innocenti—Global Office of Research and Foresight, funded by Safe Online, and delivered in partnership with local actors. The Disrupting Harm survey was designed by UNICEF Innocenti and implemented by Ipsos, who conducted a nationally representative household survey of internet‐using adolescents aged 12–17 and one of their caregivers in Ethiopia, Kenya, Mozambique, Namibia, Tanzania, Uganda, Cambodia, Indonesia, Malaysia, Philippines, Thailand, and Vietnam. Adolescents were eligible if they had used the Internet at least once in the past 3 months. Fieldwork ran from January 2020 to May 2021. The target sample per country was 1000 adolescents with one parent/carer. Random probability sampling produced nationally representative estimates, with all but two countries achieving ≥95% coverage (Table S1). The survey used a mixture of Computer Assisted Personal Interviewing and Computer Assisted Self Completion. The mental health section in the adolescent survey was self‐completed using a tablet.

UNICEF Innocenti and ECPAT International obtained ethical approval from HML IRB (Health Media Lab Institutional Review Board) Research and Ethics and national‐level review boards (Table S2). Both adolescents and parents/carers provided signed informed consent.

### Participants

Data from 12,169 adolescents were initially included. Overall, 257 participants were subsequently removed due to missing data in the weighting variable, resulting in a final sample of 11,912 participants (Table 1). Of these, 5780 (48.5%) were girls, 6132 (51.5%) were boys, and mean age was 14.9 years (SD *=* 1.7). Household food security was used as a poverty indicator and measured using the item *‘Do you have enough food to eat each day?’* (Rees et al., 2020). Response options were: 0 = ‘Never’, 1 = ‘Sometimes’, 2 = ‘Often’, 3 = ‘Always’, as well as ‘Don't know’ and ‘Prefer not to say’, which were recoded as Not Available (NA).

**TABLE 1:** Socio‐demographic characteristics of the sample.

Country	*n*	Age *M* (SD)	Gender, % females (*n*)	Severe food insecurity^a^, %(*n*)
Cambodia	992	14.4 (1.7)	49 (486)	4.9 (49)
Ethiopia	1000	15.9 (1.4)	33.7 (337)	2.3 (23)
Indonesia	995	14.5 (1.7)	55.1 (548)	3.2 (32)
Kenya	1014	14.9 (1.7)	53.2 (539)	3.5 (35)
Malaysia	995	14.3 (1.7)	47.7 (475)	2.5 (25)
Mozambique	999	15.4 (1.6)	45.1 (451)	8.7 (87)
Namibia	994	15.2 (1.7)	50 (497)	7.1 (71)
Philippines	950	14.5 (1.7)	55.5 (527)	2.8 (27)
Tanzania	996	15.3 (1.6)	42.5 (423)	5.4 (54)
Thailand	967	14.4 (1.6)	57.4 (555)	5.8 (56)
Uganda	1016	15.8 (1.5)	41.8 (425)	5 (51)
Vietnam	994	14.2 (1.6)	52 (517)	1.8 (18)

Abbreviations: *M*, mean; SD, standard deviation.

^a^ Severe food insecurity: participants who answered ‘Never’ to ‘Do you have enough food to eat each day?’.

### Mental health outcome measures

Life satisfaction was measured using Cantril's ladder (Cantril, 1965; Daly, 2022; Helliwell et al., 2022). Respondents rated their current life on a scale ranging from 0 (worst possible life) to 10 (best possible life).

Psychological well‐being was measured using a 5‐point scale version of the Children’s Worlds Psychological Well‐Being Scale (CW‐PWBS; Rees & Main, 2015; Rees et al., 2020), comprising 6 items: ‘*I like being the way I am*’, ‘*I am good at managing my daily responsibilities*’, ‘*People are generally friendly towards me*’, ‘*I have enough choice about how I spend my time*’, ‘*I feel that I am learning a lot at the moment*’, and ‘*I feel positive about my future*’. Response options were: 0 = ‘Strongly disagree’, 1 = ‘Disagree’, 2 = ‘Neither agree nor disagree’, 3 = ‘Agree’, and 4 = ‘Strongly agree’. A total score (0–24) was calculated by summing all item scores, with higher scores indicating greater well‐being.

Anxiety was measured using a 4‐item scale from the UNICEF's Youth Empowerment Project. Participants were asked to indicate the frequency with which they experienced the following in the previous 2 weeks: *‘Feel fearful’*, *‘Feel nervous’*, *‘Feel restless*, *like you can't sit still’*, and *‘Generally feel worried’*. Response options were: 0 = ‘Not at all’, 1 = ‘Rarely’, 2 = ‘Sometimes’, and 3 = ‘Often’. A total score (0–12) was computed by summing all items, with higher scores indicating greater anxiety.

Low mood/depressive symptoms and loneliness were measured by single items (‘*In the past seven days*, *how often did you feel depressed?*’; ‘*In the past seven days*, *how often did you feel lonely?’*), adapted from the Centre of Epidemiologic Studies Depression Scale (CES‐D‐10; Andresen et al., 1994; Kilburn et al., 2018). Each item was rated on a 5‐point scale, with response options recoded as: 0 = ‘Never’, and 1 = ‘1–2 days’, ‘3–4 days’, ‘5–6 days’ and ‘Every day’.

Self‐harm was assessed with one item: *‘In the past year have you hurt yourself on purpose in any way?’*. Responses (‘Yes’/‘No’) were recoded as binary (1 = ‘Yes’, 0 = ‘No’).

Suicidal ideation/attempts were measured using an adapted version from the Paykel Suicide Scale (Paykel et al., 1974). The present study included 4 of the 5 items from the scale, evaluating suicidal feelings (*‘Have you felt that life was not worth living?’*, *‘Have you thought of taking your life*, *even if you would not really do it?*’, *‘Have you reached the point where you seriously considered taking your life or perhaps made plans how you would go about doing it?’*) and suicidal attempts (*‘Have you made an attempt to take your life?’*) in the past year. Responses (‘Yes’/‘No’) were recoded as binary (1 = ’Yes', 0 = ’No’) and combined into a single indicator reflecting any ‘Yes’ response.

All scales included ‘Don't know’ and ‘Prefer not to say’ as additional response options.

### Data analyses

Data analyses were conducted using R version 4.3.2 (R Core Team, 2023) in RStudio version 2023.09.1. Scaled post‐stratification weights were applied, centrally calculated by Ipsos, including inverse probability, non‐response and post‐stratification weighting.

First, ‘Don't know’ and ‘Prefer not to say’ responses were recoded as NAs and treated as missing data. Missing data was calculated for each mental health variable by country (Table S3). Multiple imputation using multivariate imputation by chained equations (MICE) was conducted using the *mice* package (van Buuren & Groothuis‐Oudshoorn, 2011), and imputed data were used for subsequent analyses, except for Confirmatory Factor Analysis (CFA).

Secondly, CFA assessed measurement invariance of the multi‐item psychological wellbeing and anxiety scales across countries, using the *lavaan* package (Rosseel, 2012). Each scale was tested separately. Wellbeing items (5‐point scale) were treated as continuous and anxiety items (fewer than 5 points) as ordinal (Hirschfeld & von Brachel, 2014; Luong & Flake, 2023). For the CW‐PWBS, robust maximum likelihood (MLR) was used due to non‐normality (Luong & Flake, 2023), with missing data handled using full information maximum likelihood (FIML; Rosseel, 2012). For the anxiety scale, weighted least squares means and variance adjusted (WLSMV) estimation was used (Li, 2016), with pairwise deletion for missing data (Rosseel, 2012).

An initial model using the country‐pooled sample confirmed the factor structure of the scales. A multi‐group CFA tested measurement invariance across countries, using three nested models: configural invariance (equality of factor model), metric invariance (equality of factor loadings), and scalar invariance (equivalence of item intercepts/thresholds; Beaujean, 2014; Hirschfeld & von Brachel, 2014; Luong & Flake, 2023; Putnick & Bornstein, 2016). For all models, we reported: the chi‐square model fit test, the Comparative Fit Index (CFI), the Root Mean Square Error of Approximation (RMSEA), and the Standardised Root Mean Square Residual (SRMR). Excellent model fit was defined as CFI>0.95, RMSEA<0.05 and SRMR<0.05, and acceptable fit as CFI>0.90, RMSEA<0.08 and SRMR<0.08 (Hu & Bentler, 1999; Nahkur & Casas, 2021). Additionally, the next level of invariance was not supported if CFI decreased by more than 0.01, RMSEA increased by more than 0.15, or SRMR increased by more than 0.30 for metric invariance or 0.15 for scalar invariance (Chen, 2007). Mozambique was not included in the CFA for the CW‐PWBS due to the high proportion (43%) of missing data.

Descriptive statistics were computed using the *survey* package (Lumley, 2023), reported by country, and also disaggregated by age and gender. Medians and interquartile ranges were reported for continuous variables and frequencies and percentages for binary variables. Regression analyses explored the association of gender, age, household food security and the interaction between age and gender with each mental health outcome by country. Multiple linear regression was used for continuous variables and multivariable logistic regression for binary variables, using the *survey* package (Lumley, 2023).

## RESULTS

### Measurement invariance

The CW‐PSWBS fitted well for the initial model using the pooled data from 11 countries, and the configural model fit was also acceptable (Table 2). However, the metric model fit was not supported, because the decrease in the CFI was higher than 0.01. The anxiety scale fitted well for the pooled data from the 12 countries. However, the configural model was not supported, as RMSEA was higher than 0.08.

**TABLE 2:** CFA fit statistics.

Model	χ^2^(df)	*p*‐value	CFI_r_	RMSEA_r_ (CI 90%)	SRMR	ΔCFI_r_	ΔRMSEA_r_	ΔSRMR
CW‐PSWBS								
Initial	113.81 (9)	<0.001	0.970	0.051 (0.043–0.06)	0.022	‐	‐	‐
Configural	302.53 (99)	<0.001	0.962	0.066 (0.057–0.075)	0.031	‐	‐	‐
Metric	425.4 (149)	<0.001	0.948	0.062 (0.055–0.069)	0.049	0.014	0.003	0.018
Scalar	1409.86 (199)	<0.001	0.796	0.107 (0.102–0.113)	0.086	0.152	0.045	0.037

Model	χ^2^(df)	*p*‐value	CFI_s_	RMSEA_s_ (CI 90%)	SRMR	ΔCFI_s_	ΔRMSEA_s_	ΔSRMR
Anxiety scale								
Initial	79.45 (2)	<0.001	0.998	0.057 (0.047–0.068)	0.01	‐	‐	‐
Configural	206.08 (24)	<0.001	0.998	0.088 (0.077–0.099)	0.01	‐	‐	‐
Metric	478.54 (57)	<0.001	0.995	0.087 (0.08–0.094)	0.02	0.003	0.001	0.01
Scalar	1251.3 (134)	<0.001	0.986	0.092 (0.087–0.097)	0.08	0.009	0.005	0.06

Abbreviations: CFI, Comparative Fit Index; CI, confidence interval; CW‐PSWBS, Children's Worlds Psychological Well‐Being Scale; df, degrees of freedom; _r_, robust; RMSEA, root mean square error of approximation; _s_, scaled; SRMR, standardised root mean square residual.

### Mental health estimates by country

**FIGURE 1:**
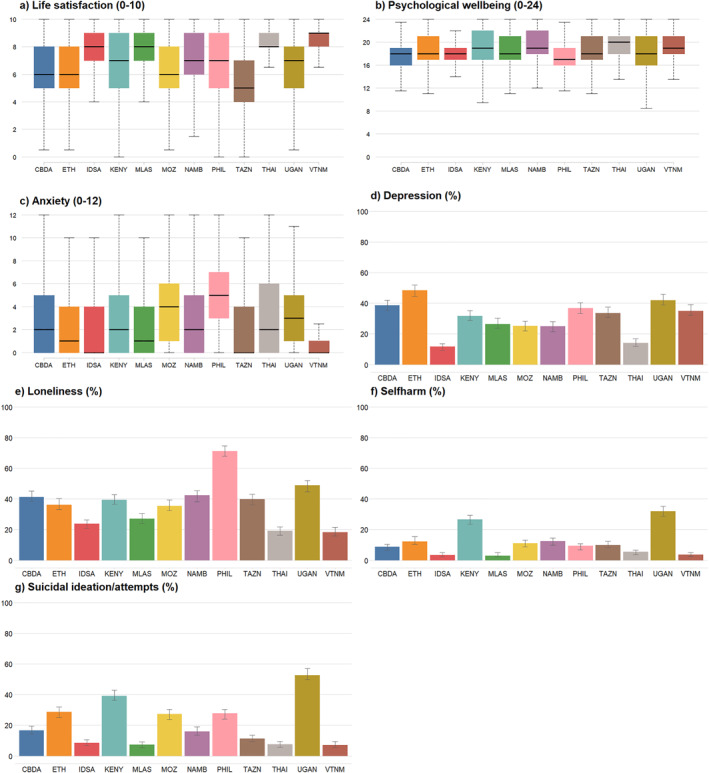
Mental health estimates by country using imputed data. Error bars in (d–g) show 95% CIs. CBDA, Cambodia; ETH, Ethiopia; IDSA, Indonesia; KENY, Kenya; MLAS, Malaysia; MOZ, Mozambique; NAMB, Namibia; PHIL, Philippines; TAZN, Tanzania; THAI, Thailand; UGAN, Uganda.

No consistent pattern emerged for the mental health estimates across countries (Table 3, Figure 1, Table S4), with large variations observed. Loneliness ranged from 18.3% (confidence interval [CI] 95%: 15.9–21.5; Vietnam) to 71.3% (CI 95%: 68–74.6; Philippines) across all countries and Asian countries, and from 35.4% (CI 95%: 32.4–39.3; Mozambique) to 48.9% (CI 95%: 44.8–52; Uganda) in African countries. Suicidal ideation/attempts ranged from 7% (CI 95%: 5.6–9.3; Vietnam) to 52.7% (CI 95%: 49.9–57; Uganda) across all countries; from 11.3% (CI 95%: 9.3–13.6; Tanzania) to 52.7% (Uganda) in African countries; and from 7% (Vietnam) to 27.8% (CI 95%: 24–30.4; Philippines) in Asian countries. Depression ranged from 11.7% (CI 95%: 9.2–13.5; Indonesia) to 48.4% (CI 95%: 44.4–51.8; Ethiopia) across all countries; from 24.9% (CI 95%: 21.5–28.0; Namibia) to 48.4% (Ethiopia) in African countries; and from 11.7% (Indonesia) to 38.6% (CI 95%: 35.3–41.9; Cambodia) in Asian countries. Self‐harm ranged from 3% (CI 95%: 2.1–4.9; Malaysia) to 32% (CI 95%: 28.7–35.3; Uganda) across all countries; from 9.9% (CI 95%: 8.2–12.4; Tanzania) to 32% (Uganda) in African countries; and from 3% (Malaysia) to 9.5% (CI 95%: 6.8–10.9; Philippines) in Asian countries. Median anxiety ranged from 0 (IQR = 0–1; Vietnam) to 5 (IQR = 3–7; Philippines) across all countries and Asian countries, and from 0 (IQR = 0–4; Tanzania) to 4 (IQR = 1–6; Mozambique) in African countries. Median life satisfaction ranged from 5 (IQR = 4–7; Tanzania) to 9 (IQR = 8–9; Vietnam) across all countries; from 5 (Tanzania) to 7 (IQR = 6–9; Namibia) in African countries; and from 6 (IQR = 5–8; Cambodia) to 9 (Vietnam) in Asian countries. Finally, median psychological wellbeing ranged from 17 (IQR = 16–19; Philippines) to 20 (IQR = 18–21; Thailand) across all countries and Asian countries, and from 18 (IQR = 16–21; Uganda) to 19 (IQR = 18–22; Namibia) in African countries.

**TABLE 3:** Mental health estimates by country using imputed data.

Country	*n* ^a^	Life satisfaction Mdn [Q1–Q3]	Psychological wellbeing Mdn [Q1–Q3]	Anxiety Mdn [Q1–Q3]	Depression %(*n*)	Loneliness %(*n*)	Self‐harm %(*n*)	Suicidal ideation/attempts %(*n*)
Cambodia	992	6 [5–8]	18 [16–19]	2 [0–5]	38.6 (383)	41.3 (410)	8.7 (86)	16.6 (164)
Ethiopia	1000	6 [5–8]	18 [17–21]	1 [0–4]	48.4 (484)	36.2 (362)	12.1 (121)	28.7 (287)
Indonesia	995	8 [7–9]	18 [17–19]	0 [0–4]	11.7 (117)	23.8 (237)	3.4 (34)	8.4 (84)
Kenya	1014	7 [5–9]	19 [17–22]	2 [0–5]	31.6 (321)	39.4 (400)	26.6 (270)	39.1 (397)
Malaysia	995	8 [7–9]	18 [17–21]	1 [0–4]	26.3 (262)	27 (269)	3 (30)	7.4 (73)
Mozambique	999	6 [5–8]	NA^b^	4 [1–6]	25.3 (253)	35.4 (354)	11.1 (111)	27.2 (272)
Namibia	994	7 [6–9]	19 [18–22]	2 [0–5]	24.9 (247)	42.4 (421)	12.4 (123)	15.9 (158)
Philippines	950	7 [5–9]	17 [16–19]	5 [3–7]	36.8 (350)	71.3 (677)	9.5 (90)	27.8 (264)
Tanzania	996	5 [4–7]	18 [17–21]	0 [0–4]	33.6 (335)	39.9 (397)	9.9 (98)	11.3 (112)
Thailand	967	8 [8–9]	20 [18–21]	2 [0–6]	14.1 (137)	19.2 (186)	5.4 (52)	7.5 (72)
Uganda	1016	7 [5–8]	18 [16–21]	3 [1–5]	42 (427)	48.9 (497)	32 (325)	52.7 (535)
Vietnam	994	9 [8–9]	19 [18–21]	0 [0–1]	34.9 (347)	18.3 (182)	3.5 (35)	7 (69)

*Note*: Mdn, median; IQR, interquartile range [Q1 first quartile–Q3 third quartile].

^a^ Non‐imputed.

^b^ Not imputed due to the proportion of missing data for psychological wellbeing in Mozambique (43%).

### Mental health estimates disaggregated by gender and age

Descriptive statistics by gender showed no consistent pattern (Tables S5 and S6). Life satisfaction medians were comparable between adolescent girls and boys across all countries. Psychological wellbeing medians were equal in six countries, but females had a one‐point advantage in Ethiopia, Tanzania, Thailand, and Vietnam, while males had a one‐point advantage in the Philippines. Anxiety medians were equal across all countries, except in Malaysia, where females had a two‐point advantage. The same proportion of males and females reported feeling depressed in Namibia, but depression estimates were higher in girls in eight countries (differences: 0.5% in Mozambique to 9.7% in Malaysia); while they were higher in boys in Kenya, Ethiopia, and Cambodia (differences: 0.3% in Kenya to 7.5% in Cambodia). Loneliness was higher in girls in eight countries (differences: 0.7% in Kenya to 6.9% in Philippines), and in boys in Cambodia, Thailand, Vietnam and Mozambique (differences: 0.3% in Mozambique to 7.8% Cambodia). The same proportion of males and females reported self‐harm in Malaysia, compared to more females in seven countries (differences: 0.1% in Vietnam to 2.8% in Ethiopia); and more males in Mozambique, Kenya, Namibia, and Cambodia (differences: 0.4% in Cambodia to 3.5% in Mozambique). Finally, suicidal ideation/attempts were higher among girls in all countries (differences: 0.4% Mozambique to 7.8% in Uganda), except Cambodia, where a between‐gender difference of 2% in favour of males was reported.

**FIGURE 2:**
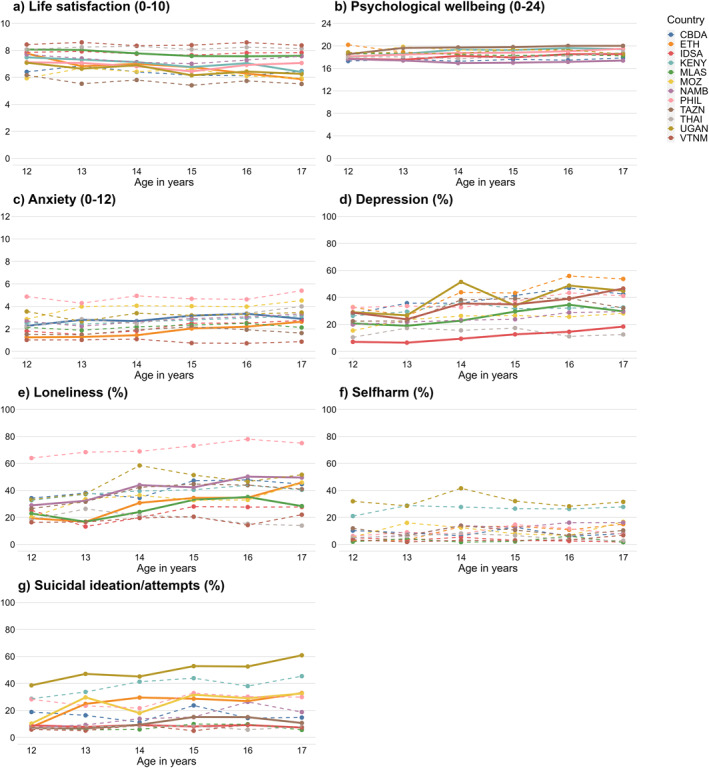
Mental health estimates by age (in years) per country using imputed data. Solid lines: significant associations with age.

Mental health estimates by age are reported in Tables S7 and S8, and illustrated in Figure 2. Life satisfaction medians were equal between younger (12–14‐year‐olds) and older (15–17‐year‐olds) adolescents in nine countries, but higher in younger adolescents in Ethiopia, Namibia, and Uganda. Psychological wellbeing medians were equal in six countries for both age groups, but higher in older adolescents in Kenya, Tanzania and Thailand, and higher in younger adolescents in Ethiopia and the Philippines. Anxiety medians were equal for both age groups in seven countries, but higher in older adolescents in Indonesia, Malaysia, Ethiopia, Namibia, and Thailand (1–4 points higher). Depression was higher in older adolescents in 11 countries (differences: 1.1% in Kenya to 16.2% in Ethiopia), but slightly higher in younger adolescents in Thailand (0.9% difference). Loneliness was higher in older adolescents in all countries (differences: 1.3% in Vietnam to 15.8% in Ethiopia), except Thailand, where more younger adolescents reported loneliness (5.5% difference). Self‐harm was higher in older adolescents in seven countries (differences: 0.6% in Cambodia to 5.9% in Namibia), but higher in younger adolescents in Uganda, Indonesia, Thailand, Tanzania, and Mozambique (differences: 1.2% in Mozambique to 4.7% in Uganda). Suicidal ideation/attempts were higher in older participants in all countries (differences: 0.5% in Vietnam to 11.6% in Uganda), except Indonesia and Thailand, were younger adolescents reported slightly higher rates (0.4% difference).

### Multivariable regression analyses of the associations between socio‐demographic characteristics and mental health estimates

Regression analyses identified significant independent associations between age, gender, food security and different mental health variables within each country, which largely reflected the bivariate analysis presented above (Tables S9 and S10). The exception was for interactions between gender and age, which were significant only for life satisfaction and psychological wellbeing in the Philippines, and for suicidal ideation/attempts in Indonesia and Kenya (Figure S1), suggesting that the association between age and these outcomes differs between males and females.

**FIGURE 3:**
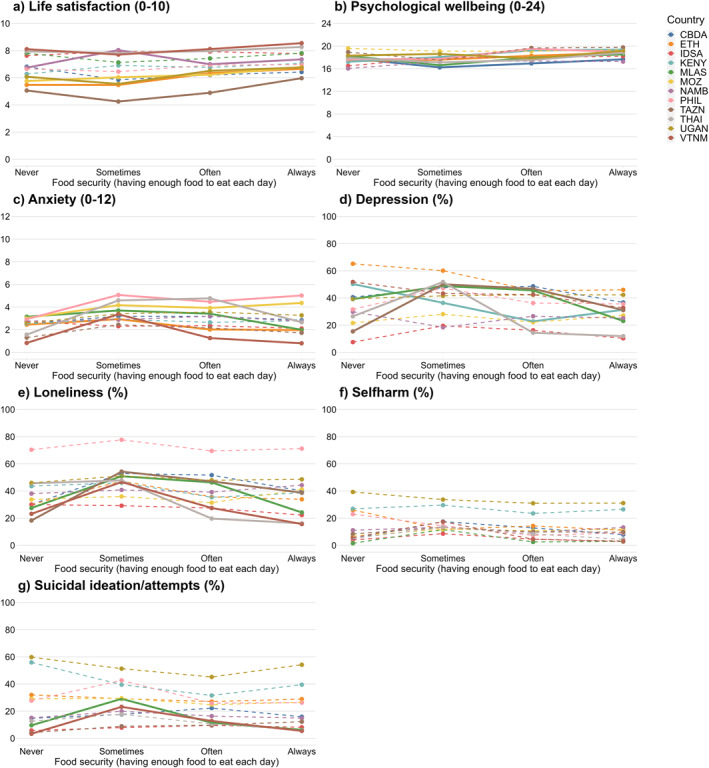
Mental health and wellbeing estimates by food insecurity per country using imputed data. Solid lines: significant associations with food security (Wald test).

Food security was significantly associated with life satisfaction in seven countries: Ethiopia (positive linear: *p* = 0.02), Mozambique (positive linear: *p* = 0.005), Namibia (quadratic: *p* = 0.02; cubic: *p* < 0.001), Tanzania (positive linear: *p* = 0.04; quadratic: *p* < 0.001), Uganda (cubic: *p* = 0.02), Thailand (no significant association for individual terms), and Vietnam (n.s.). Food security was significantly associated with psychological wellbeing in seven countries: Cambodia (quadratic: *p* = 0.001), Ethiopia (positive linear: *p* = 0.009), Kenya (positive linear: *p* = 0.003), Malaysia (quadratic: *p* = 0.021), Tanzania (positive linear: *p* = 0.004; cubic: *p* = 0.034), Uganda (quadratic: *p* = 0.003), and Vietnam (n.s.). Food security was significantly associated with anxiety in six countries: Malaysia (quadratic: *p* = 0.02), Mozambique (positive linear: *p* = 0.005; cubic: *p* = 0.02), the Philippines (positive linear: *p* = 0.01; cubic: *p* = 0.01), Thailand (positive linear: *p* = 0.05; quadratic: *p* < 0.001), Vietnam (quadratic: *p* = 0.04), and Ethiopia (n.s.). Food security was significantly associated with depression in four countries: Kenya (negative linear: *p* = 0.01; quadratic: *p* = 0.04), Malaysia (quadratic: *p* = 0.03), Tanzania (positive linear: *p* = 0.02; quadratic: *p* < 0.001), and Thailand (negative linear: *p* < 0.001; quadratic: *p* = 0.04; cubic: *p* = 0.009). Food security was significantly associated with loneliness in four countries: Malaysia (quadratic: *p* = 0.007), Tanzania (positive linear: *p* = 0.008; quadratic: *p* < 0.001), Thailand (negative linear: *p* < 0.001), and Vietnam (n.s.). Food security was significantly associated with suicidal ideation/attempts in Malaysia and Vietnam (quadratic; *p* = 0.02). No significant associations were found between food security and self‐harm (Figure 3).

## DISCUSSION

This study offers key insights into the validity and use of different mental health measures among adolescents in 12 LMICs. Cross‐measurement invariance was not supported for either the CW‐PWBS or the anxiety scale, consistent with previous studies (Casas & González‐Carrasco, 2021) and limiting meaningful cross‐country comparisons. More specifically, the metric model fit was not supported for the CW‐PSWBS, indicating that the strength of the relationships between items and the latent construct differed across countries, and that participants in different countries may interpret or respond to the items differently. In contrast, the configural model for the anxiety scale was not supported, suggesting differences in construct conceptualisation across countries. Measurement invariance was not assessed for the other measures due to their single‐item format or the use of severity measures. For these reasons, although mental health estimates were reported, these should be interpreted with caution as estimates that relate to a particular country.

Our mental health estimates suggest no consistent pattern across countries, with ranges varying from small to large depending on the outcome. The greatest variation was in reports of loneliness, ranging from 18.3% in Vietnam to 71.3% in the Philippines. The Philippines also reported the highest anxiety levels and lowest psychological wellbeing. The Philippines had particularly strict COVID‐related government policies (Hale et al., 2021), which may partly explain these results. Other factors, such as high migratory separation from parents, have also been associated with adolescent loneliness in this country (Smeekens et al., 2012).

Estimate ranges by region suggested lower life satisfaction, and higher levels of depression, self‐harm and suicidal ideation/attempts in the African countries studied, consistent with previous research (Helliwell, 2024; Li et al., 2021; McKinnon et al., 2016; Uddin et al., 2019; WHO, 2021b), suggesting that these measures capture meaningful variation, although they are most appropriately applied for within‐country analyses. Suicidal ideation/attempts ranged from 11.3% to 52.7% in African countries, and from 7% to 27.8% in Asian countries. In addition to risk factors common across regions, previously cited challenges in the African region include a high burden of HIV/AIDS and infectious diseases, political tensions, violence, child marriage, adolescent pregnancy, insufficient action to prevent risk factors, inadequate investment in mental health and a lack of suicide prevention strategies and policies in many countries (Biswas et al., 2020; Li et al., 2021; Liu et al., 2018; McKinnon et al., 2016; Ongeri et al., 2023; Smith et al., 2021; Uddin et al., 2019; WHO, 2021a; WHO Regional Office for Africa, 2022). The highest estimates were in Uganda and Kenya, possibly due to strict COVID‐related policies, and concurrent crises including poverty, locust invasions, political instability and extreme weather at the time of data collection (Hale et al., 2021; Khisa & Rwengabo, 2023; UN in Kenya, 2020; World Bank). Lower reports of suicidal ideation/attempts in South‐East Asian countries have been attributed to religious and cultural factors, underreporting due to stigma, and a higher proportion of countries with suicide prevention strategies and policies (Li et al., 2021; Liu et al., 2018; McKinnon et al., 2016; Uddin et al., 2019; WHO, 2021a).

Socio‐demographic variations by age, gender, and food security were also reported. Depression, loneliness, self‐harm and suicidal ideation/attempts were higher for girls in most countries, aligning with previous findings. However, between‐gender differences varied in magnitude, and the gender gap was reversed or absent in a minority of countries, contrary to the consistent female excess observed in HICs (Campbell et al., 2021; Uddin et al., 2019). Campbell et al. found overall worse mental health in girls across 73 countries but also a reversed gender gap in a minority of countries. Our regression analyses identified a significant association with gender only for loneliness in Malaysia, life satisfaction and psychological wellbeing in the Philippines, and for suicidal ideation/attempts in Indonesia, suggesting that the measures capture expected demographic variation, but that these associations may vary across countries. Recent studies suggest gender contributes less to mental health disparities in LMICs than HICs (Liu et al., 2018). Campbell et al. found that higher GDP per capita and greater gender equality were associated with a larger mental health gender gap, possibly due to rising expectations for gender equality outpacing actual societal change. For instance, Malaysia has the highest GDP per capita and the lowest gender inequality index among the countries included in the current study, while Indonesia and Philippines are amongst the six countries with the highest GDP per capita and the lowest gender inequality index among those included (World Bank).

Depression, loneliness, self‐harm and suicidal ideation/attempts were higher among older compared to younger adolescents in most countries. Anxiety estimates were either equal or higher in older adolescents, and life satisfaction was either equal or lower in older adolescents. These differences varied in magnitude across countries and outcomes and were sometimes negligible. Regression analyses showed significant negative associations between age and life satisfaction in five countries, and with psychological wellbeing in the Philippines, while we found significant positive associations between age and anxiety in two countries, depression and suicidal ideation/attempts in four countries, and loneliness in three countries. These results, suggesting worsening life satisfaction and psychological wellbeing, along with increased levels of anxiety, depression, loneliness, and suicidal ideation/attempts with increasing age during adolescence, are consistent with previous literature and indicate that our measures capture expected age patterns (Kieling et al., 2024; Liu et al., 2018). However, regression analyses revealed that psychological wellbeing increased with age in a minority of countries, and that suicidal ideation/attempts decreased with age in Indonesia, contrary to what is consistently reported in HICs. Variability between age groups was small in these countries, showing only small fluctuations across ages.

Food security was significantly associated with life satisfaction and psychological wellbeing in seven countries, with anxiety in six countries, with depression and loneliness in four countries, and with suicidal ideation/attempts in two countries. This is consistent with previous research showing an association between food insecurity and mental health and indicates that our measures behave as expected in relation to a known correlate (Fram et al., 2022; Koyanagi et al., 2019; Shayo & Lawala, 2019; Smith et al., 2023). Overall, while measurement invariance was not supported, our regression models suggest that the mental health measures used in this study capture meaningful variation in relation to well‐established determinants of mental health, with some exceptions, supporting their construct validity across diverse settings. Additionally, our results suggest food insecurity is associated with different mental health outcomes, particularly life satisfaction and psychological wellbeing. However, associations were sometimes non‐linear, revealing complex relationships. The greater impact of relative position compared to personal socioeconomic circumstances or the Easterlin paradox might partly explain these complex associations (Campbell et al., 2021; Piera Pi‐Sunyer et al., 2023). Previous studies suggest that the association between food insecurity and mental health may be stronger in countries with lower population prevalence of food insecurity, possibly due to a greater negative impact on social status (Koyanagi et al., 2019). In line with this, Vietnam and Malaysia, two of the countries with the lowest rates of severe food insecurity, showed an association between food insecurity and five mental health outcomes. Overall, while our findings on socio‐demographic variations generally align with previous evidence, they should primarily be viewed as supporting the construct validity of the mental health measures used, rather than as evidence of causal relationships.

There are several strengths to this study. First, random probability sampling was used, enhancing sample representativeness and potentially improving the validity of cross‐country comparisons (Atilola, 2015). Secondly, multiple mental health indicators were explored, offering a more comprehensive picture of adolescent mental health. Third, the study participants were adolescents living in LMICs, which is still relatively rare in mental health research, providing an opportunity for assessing instrument performance with this particular group. Finally, at the time of data collection, legislation criminalising suicide attempts still existed in Kenya, Malaysia, Tanzania and Uganda, potentially influencing reporting (United for Global Mental Health, 2021). The highest estimates of suicidal ideation/behaviours in the present study were observed in Kenya and Uganda. Using self‐administered questionnaires might have contributed to a higher disclosure of suicidal ideation/behaviours (Rickwood & Coleman‐Rose, 2023).

There are also some limitations in this study. CFA results for the anxiety scale need to be interpreted with caution, as cut‐offs used were not originally proposed for WLSMV estimation of ordinal data. Since WLSMV estimation was used for the anxiety scale, sampling weights were not applied, possibly influencing our results. However, applying other estimation methods (e.g., PML‐pairwise maximum likelihood) yielded consistent results. Another limitation is the use of household surveys, which although it provides the opportunity for relatively anonymous reporting on sensitive topics, may have led to the exclusion of high‐risk and difficult‐to‐reach groups. Additionally, although Internet usage rates were considered in the weighting procedure, there are significant disparities in Internet connectivity between HICs and LMICs (UNICEF and International Telecommunication Union, 2020). The inclusion of only internet‐using adolescents may have affected the representativeness of our population. Single‐item measures represent a limitation, due to their lack of precision, construct validity, and lower reliability, and they limit the evaluation of measurement invariance. Our measures capture typical symptoms, but do not constitute formal psychiatric diagnoses, and results should not be interpreted as epidemiological prevalence estimates. Suicide item responses were recoded into a single total binary indicator for ‘yes’ on any item, possibly contributing to higher observed rates of suicide‐related outcomes, and future studies are encouraged to report suicidal ideation/attempts separately as well as combined. Finally, the use of a cross‐sectional survey did not allow for the exploration of causal effects between potential predictors and mental health outcomes.

## CONCLUSION

In conclusion, our findings offer key insights into the validity and use of a range of mental health measures in the adolescent population across 12 LMICs. Despite the lack of measurement invariance in these scales, our results still provided valuable within‐country insights that can help tailor research on adolescent mental health to local contexts. No consistent pattern of mental health estimates was observed across countries, and while gender and age disparities were present, their magnitude and direction varied, highlighting the importance of contextual research and nationally validated indicators. Food insecurity emerged as the most consistent correlate of mental health outcomes, in line with previous literature. This indicates additional support for existing research suggesting that interventions targeting food insecurity in adolescents may also improve their mental health. However, our findings on socio‐demographic variations should be interpreted with caution and primarily be viewed as supporting the construct validity of the mental health measures used. Importantly, further research is needed to better understand the risk and protective factors influencing these mental health outcomes, and to support the development and evaluation of cross‐country indicators that can help us better measure adolescent mental health in LMICs.

## AUTHOR CONTRIBUTIONS

**Ariadna Albajara Sáenz**: Conceptualization; data curation; formal analysis; methodology; project administration; visualization; writing—original draft preparation. **Sebastian Kurten**: Conceptualization; data curation; formal analysis; methodology; writing—review and editing. **Jennifer Saxton**: Conceptualization; methodology; writing—review and editing. **Daniel Kardefelt‐Winther**: Conceptualization; data curation; investigation; methodology; project administration; writing—review and editing. **Tamsin Ford**: Conceptualization; methodology; project administration; supervision; writing—review and editing. **Amy Orben**: Conceptualization; methodology; project administration; supervision; writing—review and editing. **Simon R. White**: Data Curation; formal analysis; methodology; supervision; writing—review and editing.

## CONFLICT OF INTEREST STATEMENT

Tamsin Ford's research group receives payment for research methods consultancy from Place2Be. This work was not related to this manuscript. The remaining authors have declared that they have no competing or potential conflicts of interest.

## ETHICAL CONSIDERATIONS

This study involved secondary analysis of previously collected data and did not require ethical approval.

## Supporting information

Supporting Information S1

## Data Availability

The original data used cannot be shared by the authors at this moment.
