# Supplementary material for: Mental health measures among adolescents in 12 low‐ and middle‐income countries: Measurement invariance and cross‐sectional analyses of Disrupting Harm survey data
Source: JCPP Adv. 2025 Dec 13:e70087. Online ahead of print. doi: 10.1002/jcv2.70087 (PMC13338989; doi:10.1002/jcv2.70087)
Supplement: Supplementary file 1 — Supporting Information S1 [file JCV2-9999-e70087-s001.docx]

**Mental health measures among adolescents in 12 low- and middle-income countries: Measurement invariance and cross-sectional analyses of Disrupting Harm survey data**

**Supporting Information**

**Table S1. Fieldwork dates, coverage and achieved sample size per country.**

| **Country** | **Fieldwork start date** | **Fieldwork end date** | **Fieldwork coverage (%)** | **Achieved sample size** |
| --- | --- | --- | --- | --- |
| Cambodia | 10/11/2020 | 31/12/2020 | 100 | 1007 |
| Ethiopia | 21/12/2020 | 19/01/2021 | 82 | 1021 |
| Indonesia | 26/11/2020 | 28/02/2021 | 76 | 1012 |
| Kenya | 21/12/2020 | 19/01/2021 | 100 | 1016 |
| Malaysia | 12/04/2021 | 05/11/2021 | 94-97 | 1009 |
| Mozambique | South: 17/02/2021;  Central and North: 13/05/2021 | 30/07/2021 | 100 | 1111 |
| Namibia | 21/12/2020 | 28/02/2021 | 100 | 1000 |
| Philippines | 11/01/2021 | 15/04/2021 | 96 | 975 |
| Tanzania | 21/12/2020 | 19/01/2021 | 99 | 1000 |
| Thailand | 21/11/2020 | 23/02/2021 | 98 | 1000 |
| Uganda | 04/01/2020 | 19/01/2021 | 96 | 1020 |
| Vietnam | 21/11/2020 | 22/02/2021 | 95 | 998 |

Fieldwork coverage: proportion of the total population that had a chance of being included in the survey sample.

**Table S2. Ethical review boards by country.**

| **Country** | **Organisation** |
| --- | --- |
| Cambodia | NA |
| Ethiopia | Ethiopian Society of Sociologists, Social Workers and Anthropologists |
| Indonesia | Health Research Ethics Committee, National Institute of Health Research and Development (HREC-NHRD) |
| Kenya | AMREF Health Africa – Science and Ethical Review Committee |
|  | National Commission for Science, Technology and Innovation |
| Malaysia | National Medical Research Register/ Medical Research Ethics Committee |
| Mozambique | National Committee on Bioethics for Health, Ministry of health |
| Namibia | Ministry of Health and Social Services Ethical Review Board |
| Philippines | Philippine Social Science Council Ethical Review Board (SSERB) |
| Tanzania | National Institute for Medical Research |
|  | Zanzibar Health Research Institute |
|  | National Bureau of Statistics |
|  | TAMISEMI (President’s Office) |
|  | Tanzania Commission for Science and Technology (COSTECH) |
| Thailand | Mahidol University, The Institute of Human Rights and Peace studies |
| Uganda | Makerere University School of Public Health |
|  | Uganda National Council for Science and Technology |
| Vietnam | NA |

**Table S3. Proportion of missing data by country and mental health variable.**

| **Country** | **n** | **Life satisfaction**  %(n) | **Psychological wellbeing**  %(n) | **Anxiety**  %(n) | **Depression**  %(n) | **Loneliness**  %(n) | **Self-harm**  %(n) | **Suicidal ideation/ attempts**  %(n) |
| --- | --- | --- | --- | --- | --- | --- | --- | --- |
| Cambodia | 992 | 3 (30) | 6.8 (67) | 1.6 (16) | 11.2 (111) | 11.7 (116) | 6.9 (68) | 18.4 (183) |
| Ethiopia | 1000 | 1.9 (19) | 5.9 (59) | 3.6 (36) | 7.5 (75) | 9.2 (92) | 16.4 (164) | 20.5 (205) |
| Indonesia | 995 | 1.7 (17) | 4.7 (47) | 4.3 (43) | 13.8 (137) | 14 (139) | 3.1 (31) | 6.6 (66) |
| Kenya | 1014 | 1.2 (12) | 2.6 (26) | 2 (20) | 11.3 (115) | 9.1 (92) | 5.6 (57) | 7.7 (78) |
| Malaysia | 995 | 2.2 (22) | 9.9 (99) | 5.6 (56) | 8.3 (83) | 9.8 (98) | 4.3 (43) | 5.2 (52) |
| Mozambique | 999 | 8 (80) | 43 (430) | 11.1 (111) | 10.6 (106) | 6.6 (66) | 4.6 (46) | 4.7 (47) |
| Namibia | 994 | 1.1 (11) | 8.7 (86) | 6.2 (62) | 16.5 (164) | 15.2 (151) | 4.9 (49) | 4.1 (41) |
| Philippines | 950 | 3.3 (31) | 14.6 (139) | 8.5 (81) | 21.5 (204) | 19.3 (183) | 8.9 (85) | 17.7 (168) |
| Tanzania | 996 | 1.7 (17) | 1.9 (19) | 2.1 (21) | 8.2 (82) | 7.5 (75) | 2.6 (26) | 4.3 (43) |
| Thailand | 967 | 1.1 (11) | 5.4 (52) | 1.3 (13) | 1.9 (18) | 1.3 (13) | 2.9 (28) | 3 (29) |
| Uganda | 1016 | 2.3 (23) | 5.3 (54) | 4.5 (46) | 13.8 (140) | 9.4 (95) | 5.9 (60) | 6.6 (67) |
| Vietnam | 994 | 0.4 (4) | 7.2 (72) | 3.1 (31) | 5.3 (53) | 7 (70) | 2.1 (21) | 3.8 (38) |

**Table S4. Mental health and wellbeing estimates by country with non-imputed data.**

| **Country** | **n** | **Life satisfaction**  Mdn [Q1-Q3] | **Psychological wellbeing**  Mdn [Q1-Q3] | **Anxiety**  Mdn [Q1-Q3] | **Depression**  %(n) | **Loneliness**  %(n) | **Self-harm**  %(n) | **Suicidal/ideation attempts**  %(n) |
| --- | --- | --- | --- | --- | --- | --- | --- | --- |
| Cambodia | 992 | 6 [5-8] | 18 [16-19] | 2 [0-5] | 37.8 (333) | 41.4 (363) | 7.7 (72) | 17.9 (147) |
| Ethiopia | 1000 | 6 [5-8] | 19 [17-21] | 1 [0-4] | 48 (443) | 35.8 (327) | 10.3 (86) | 30.6 (247) |
| Indonesia | 995 | 8 [7-9] | 18 [17-19] | 0 [0-4] | 10.7 (92) | 22.4 (191) | 3.1 (30) | 8.5 (79) |
| Kenya | 1014 | 7 [5-9] | 19 [17-22] | 2 [0-5] | 30.3 (271) | 38.8 (357) | 26.2 (250) | 40.9 (381) |
| Malaysia | 995 | 8 [7-9] | 18 [17-21] | 0 [0-4] | 25 (229) | 25.6 (231) | 2.5 (24) | 6.9 (65) |
| Mozambique | 999 | 6 [5-8] | 18 [15-19] | 4 [1-6] | 25 (222) | 35.3 (326) | 11 (104) | 27.8 (263) |
| Namibia | 994 | 7 [6-9] | 19 [18-22] | 2 [0-5] | 24.6 (209) | 42.1 (362) | 12.1 (114) | 16.1 (154) |
| Philippines | 950 | 7 [5-9] | 17 [16-19] | 5 [3-7] | 35.9 (268) | 72 (557) | 8.9 (77) | 30.3 (232) |
| Tanzania | 996 | 5 [4-7] | 18 [17-21] | 0 [0-4] | 33.6 (308) | 40.2 (370) | 9.8 (96) | 11.4 (109) |
| Thailand | 967 | 8 [8-9] | 20 [18-21] | 2 [0-6] | 13.6 (129) | 18.8 (180) | 4.8 (46) | 7.2 (68) |
| Uganda | 1016 | 7 [5-8] | 18 [16-21] | 3 [0-5] | 41.3 (363) | 48.5 (452) | 31.6 (302) | 54.3 (517) |
| Vietnam | 994 | 9 [8-9] | 19 [18-21] | 0 [0-1] | 34.6 (324) | 17.1 (157) | 3.3 (32) | 6.4 (61) |

Mdn: median, IQR: interquartile range [Q1 first quartile - Q3 third quartile].

**Table S5. Mental health estimates by gender and country with imputed data.**

| **Gender** | **n^a^** | **Life satisfaction**  Mdn [Q1-Q3] | **Psychological wellbeing**  Mdn [Q1-Q3] | **Anxiety**  Mdn [Q1-Q3] | **Depression**  %(n) | **Loneliness**  %(n) | **Self-harm**  %(n) | **Suicidal ideation/**  **attempts**  %(n) |
| --- | --- | --- | --- | --- | --- | --- | --- | --- |
| ***Cambodia*** | | | | | | | | |
| Female | 486 | 6 [5-8] | 18 [16-19] | 2 [0-5] | 34.7 (164) | 37.2 (176) | 8.5 (40) | 15.5 (73) |
| Male | 506 | 6 [5-8] | 18 [16-19] | 2 [0-4] | 42.2 (219) | 45 (233) | 8.9 (46) | 17.5 (91) |
| ***Ethiopia*** | | | | | | | | |
| Female | 337 | 6 [5-8] | 19 [17-21] | 1 [0-4] | 47.3 (171) | 37.9 (137) | 13.9 (50) | 32.7 (118) |
| Male | 663 | 6 [5-8] | 18 [17-21] | 1 [0-4] | 49 (313) | 35.2 (225) | 11.1 (71) | 26.4 (169) |
| ***Indonesia*** | | | | | | | | |
| Female | 548 | 8 [7-9] | 18 [17-19] | 0 [0-4] | 12.9 (70) | 25.9 (141) | 3.5 (19) | 9.8 (54) |
| Male | 447 | 8 [7-9] | 18 [17-19] | 0 [0-4] | 10.2 (46) | 21.4 (96) | 3.2 (15) | 6.7 (30) |
| ***Kenya*** | | | | | | | | |
| Female | 539 | 7 [5-9] | 19 [17-22] | 2 [0-5] | 31.5 (168) | 39.8 (212) | 25.7 (137) | 41.8 (223) |
| Male | 475 | 7 [5-8] | 19 [17-21] | 2 [0-4] | 31.8 (153) | 39.1 (188) | 27.6 (133) | 36.2 (174) |
| ***Malaysia*** | | | | | | | | |
| Female | 475 | 8 [7-9] | 18 [17-20] | 2 [0-5] | 31.4 (150) | 30.3 (145) | 3 (14) | 9.5 (46) |
| Male | 520 | 8 [7-9] | 18 [17-21] | 0 [0-3] | 21.7 (112) | 24.1 (124) | 3 (16) | 5.4 (28) |
| ***Mozambique*** | | | | | | | | |
| Female | 451 | 6 [5-8] | NA* | 4 [1-6] | 25.6 (122) | 35.3 (168) | 9.3 (45) | 27.4 (131) |
| Male | 548 | 6 [5-8] | NA* | 4 [1-6] | 25.1 (131) | 35.6 (186) | 12.8 (67) | 27 (141) |
| ***Namibia*** | | | | | | | | |
| Female | 497 | 7 [6-9] | 19 [18-23] | 2 [0-5] | 24.9 (124) | 43.4 (216) | 12 (60) | 16.5 (82) |
| Male | 497 | 7 [6-9] | 19 [18-21] | 2 [0-5] | 24.9 (124) | 41.4 (205) | 12.7 (63) | 15.3 (76) |
| ***Philippines*** | | | | | | | | |
| Female | 527 | 7 [5-9] | 17 [16-19] | 5 [3-7] | 39 (206) | 74.4 (394) | 10 (53) | 30 (159) |
| Male | 423 | 7 [5-9] | 18 [16-19] | 5 [2-7] | 34.1 (143) | 67.5 (284) | 8.8 (37) | 24.9 (105) |
| ***Tanzania*** | | | | | | | | |
| Female | 423 | 5 [5-7] | 19 [17-21] | 0 [0-4] | 35.5 (154) | 41.3 (179) | 10.9 (47) | 14.4 (62) |
| Male | 573 | 5 [4-7] | 18 [17-21] | 0 [0-4] | 32.2 (181) | 38.7 (218) | 9.1 (51) | 8.9 (50) |
| ***Thailand*** | | | | | | | | |
| Female | 555 | 8 [8-9] | 20 [18-21] | 2 [0-6] | 14.9 (83) | 17.9 (100) | 6.1 (34) | 8.7 (49) |
| Male | 412 | 8 [8-9] | 19 [18-21] | 2 [0-6] | 13 (54) | 21 (86) | 4.4 (18) | 5.8 (24) |
| ***Uganda*** | | | | | | | | |
| Female | 425 | 7 [5-8] | 18 [16-21] | 3 [1-6] | 46.6 (207) | 51.1 (227) | 32.3 (143) | 57.1 (254) |
| Male | 591 | 7 [5-8] | 18 [16-21] | 3 [0-5] | 38.4 (220) | 47.2 (270) | 31.8 (182) | 49.3 (282) |
| ***Vietnam*** | | | | | | | | |
| Female | 517 | 9 [8-9] | 19 [18-21] | 0 [0-1] | 35.2 (189) | 17.3 (93) | 3.6 (19) | 7.8 (42) |
| Male | 477 | 9 [8-9] | 18 [18-21] | 0 [0-1] | 34.5 (157) | 19.5 (89) | 3.5 (16) | 6 (27) |

Mdn: median, IQR: interquartile range [Q1 first quartile - Q3 third quartile]. *Missing data for wellbeing items in Mozambique were not imputed due to the proportion of missing data for the psychological wellbeing total score (43%). ^a^Non-imputed n.

**Table S6. Mental health by gender and country with non-imputed data.**

| **Gender** | **n** | **Life satisfaction**  Mdn [Q1-Q3] | **Psychological wellbeing**  Mdn [Q1-Q3] | **Anxiety**  Mdn [Q1-Q3] | **Depression**  %(n) | **Loneliness**  %(n) | **Self-harm**  %(n) | **Suicidal ideation/ attemps**  %(n) |
| --- | --- | --- | --- | --- | --- | --- | --- | --- |
| ***Cambodia*** | | | | | | | | |
| Female | 486 | 6 [5-8] | 18 [16-19] | 2 [0-5] | 33.6 (143) | 36.8 (157) | 7.8 (35) | 16.2 (65) |
| Male | 506 | 6 [5-8] | 18 [16-19] | 2 [0-4] | 41.6 (191) | 45.7 (207) | 7.7 (36) | 19.6 (82) |
| ***Ethiopia*** | | | | | | | | |
| Female | 337 | 6 [5-8] | 19 [17-21] | 1 [0-4] | 45.9 (145) | 38 (121) | 12.1 (38) | 35.6 (104) |
| Male | 663 | 6 [5-8] | 18 [17-21] | 1 [0-4] | 49.1 (298) | 34.6 (206) | 9.2 (48) | 27.8 (143) |
| ***Indonesia*** | | | | | | | | |
| Female | 548 | 8 [7-9] | 18 [17-19] | 0 [0-4] | 12.1 (58) | 24.5 (115) | 3.3 (18) | 9.9 (51) |
| Male | 447 | 8 [7-9] | 18 [17-19] | 0 [0-3] | 8.9 (34) | 19.8 (76) | 3 (13) | 6.7 (28) |
| ***Kenya*** | | | | | | | | |
| Female | 539 | 7 [5-9] | 19 [17-22] | 2 [0-5] | 29.8 (137) | 39.2 (186) | 25.3 (127) | 43.8 (214) |
| Male | 475 | 7 [5-8] | 19 [17-21] | 2 [0-4] | 30.8 (134) | 38.4 (171) | 27.3 (123) | 37.6 (167) |
| ***Malaysia*** | | | | | | | | |
| Female | 475 | 8 [7-9] | 18 [17-20] | 2 [0-4] | 29.9 (131) | 28.1 (124) | 2.4 (11) | 9.1 (41) |
| Male | 520 | 8 [7-9] | 18 [17-21] | 0 [0-3] | 20.5 (97) | 23.2 (108) | 2.6 (13) | 4.9 (24) |
| ***Mozambique*** | | | | | | | | |
| Female | 451 | 6 [5-8] | 18 [15-19] | 4 [1-6] | 25.2 (107) | 34.9 (155) | 9.4 (42) | 28.2 (126) |
| Male | 548 | 6 [5-8] | 18 [16-19] | 3 [0-6] | 24.8 (114) | 35.6 (172) | 12.5 (62) | 27.5 (138) |
| ***Namibia*** | | | | | | | | |
| Female | 497 | 7 [6-9] | 19 [18-23] | 2 [0-5] | 24.8 (106) | 43.5 (186) | 11.5 (55) | 16.5 (80) |
| Male | 497 | 7 [6-9] | 19 [18-22] | 2 [0-5] | 24.4 (103) | 40.8 (177) | 12.7 (60) | 15.7 (74) |
| ***Philippines*** | | | | | | | | |
| Female | 527 | 7 [5-8] | 17 [16-19] | 5 [3-7] | 38.5 (155) | 75.5 (326) | 9.2 (45) | 32.2 (139) |
| Male | 423 | 7 [5-9] | 18 [15-19] | 4 [2-6] | 32.7 (113) | 67.6 (231) | 8.5 (33) | 27.9 (92) |
| ***Tanzania*** | | | | | | | | |
| Female | 423 | 5 [5-7] | 19 [17-21] | 0 [0-4] | 35.5 (138) | 41.9 (165) | 10.8 (46) | 14.6 (61) |
| Male | 573 | 5 [4-7] | 18 [17-21] | 0 [0-4] | 32.2 (170) | 38.9 (205) | 9 (49) | 8.9 (48) |
| ***Thailand*** | | | | | | | | |
| Female | 555 | 8 [8-9] | 20 [18-21] | 2 [0-6] | 14.5 (79) | 17.7 (97) | 5.5 (30) | 8.7 (47) |
| Male | 412 | 8 [8-9] | 19 [18-21] | 2 [0-6] | 12.3 (49) | 20.4 (83) | 3.9 (16) | 5.2 (21) |
| ***Uganda*** | | | | | | | | |
| Female | 425 | 7 [5-8] | 19 [17-21] | 3 [1-5] | 45.8 (177) | 50.7 (206) | 32.1 (133) | 59.5 (247) |
| Male | 591 | 7 [5-8] | 18 [16-21] | 2 [0-5] | 37.7 (186) | 46.8 (246) | 31.3 (170) | 50.3 (270) |
| ***Vietnam*** | | | | | | | | |
| Female | 517 | 9 [8-9] | 19 [18-21] | 0 [0-1] | 34.7 (172) | 16.2 (81) | 3.1 (17) | 7.1 (37) |
| Male | 477 | 9 [8-9] | 18 [18-21] | 0 [0-1] | 34.5 (152) | 18.2 (76) | 3.4 (15) | 5.5 (24) |

Mdn: median, IQR: interquartile range [Q1 first quartile - Q3 third quartile].

**Table S7. Mental health and wellbeing estimates by age and country with imputed data.**

| **Age** | **n^a^** | **Life satisfaction**  Mdn [Q1-Q3] | **Psychological wellbeing**  Mdn [Q1-Q3] | **Anxiety**  Mdn [Q1-Q3] | **Depression**  %(n) | **Loneliness**  %(n) | **Self-harm**  %(n) | **Suicidal ideation/ attempts**  %(n) |
| --- | --- | --- | --- | --- | --- | --- | --- | --- |
| ***Cambodia*** | | | | | | | | |
| 12-14 | 535 | 6 [5-8] | 18 [16-19] | 2 [0-4] | 33.2 (158) | 35.6 (170) | 8.4 (40) | 15.4 (73) |
| 15-17 | 457 | 6 [5-8] | 18 [16-19] | 2 [0-5] | 43.6 (224) | 46.6 (240) | 9 (46) | 17.6 (91) |
| ***Ethiopia*** | | | | | | | | |
| 12-14 | 185 | 7 [5-10] | 19 [17-21] | 0 [0-2] | 36.2 (90) | 24.3 (60) | 8.1 (20) | 24.3 (60) |
| 15-17 | 815 | 6 [5-8] | 18 [16-21] | 2 [0-4] | 52.4 (394) | 40.1 (301) | 13.4 (101) | 30.2 (227) |
| ***Indonesia*** | | | | | | | | |
| 12-14 | 496 | 8 [7-9] | 18 [17-19] | 0 [0-3] | 7.7 (36) | 19.3 (90) | 4.5 (21) | 8.6 (40) |
| 15-17 | 499 | 8 [7-9] | 18 [17-19] | 1 [0-5] | 15.2 (81) | 27.8 (148) | 2.4 (13) | 8.2 (44) |
| ***Kenya*** | | | | | | | | |
| 12-14 | 420 | 7 [6-9] | 19 [17-21] | 2 [0-4] | 31 (140) | 36.7 (166) | 26.2 (118) | 34.9 (157) |
| 15-17 | 594 | 7 [5-8] | 20 [18-22] | 2 [0-5] | 32.1 (181) | 41.6 (234) | 26.9 (152) | 42.5 (239) |
| ***Malaysia*** | | | | | | | | |
| 12-14 | 535 | 8 [7-9] | 18 [17-21] | 0 [0-4] | 20.8 (98) | 21.3 (100) | 2.6 (12) | 6.2 (29) |
| 15-17 | 460 | 8 [7-9] | 18 [17-20] | 1 [0-4] | 31.3 (164) | 32.2 (169) | 3.3 (17) | 8.4 (44) |
| ***Mozambique*** | | | | | | | | |
| 12-14 | 262 | 6 [5-8] | NA* | 4 [1-6] | 22.6 (79) | 31.4 (110) | 11.9 (42) | 20.1 (70) |
| 15-17 | 737 | 6 [5-8] | NA* | 4 [1-7] | 26.8 (174) | 37.6 (244) | 10.7 (69) | 31 (201) |
| ***Namibia*** | | | | | | | | |
| 12-14 | 300 | 8 [6-9] | 19 [18-22] | 1 [0-5] | 21.8 (95) | 36 (157) | 9.1 (40) | 10.6 (46) |
| 15-17 | 694 | 7 [6-9] | 19 [18-22] | 3 [0-5] | 27.3 (152) | 47.3 (264) | 15 (83) | 20 (112) |
| ***Philippines*** | | | | | | | | |
| 12-14 | 489 | 7 [5-9] | 18 [16-19] | 5 [3-7] | 32.9 (154) | 67.1 (314) | 7.6 (36) | 24.5 (114) |
| 15-17 | 461 | 7 [5-8] | 17 [16-19] | 5 [3-7] | 40.6 (196) | 75.4 (364) | 11.3 (55) | 31 (149) |
| ***Tanzania*** | | | | | | | | |
| 12-14 | 319 | 5 [5-7] | 18 [16-21] | 0 [0-3] | 29.1 (117) | 35 (140) | 10.8 (43) | 7.9 (32) |
| 15-17 | 677 | 5 [4-7] | 19 [17-22] | 0 [0-4] | 36.7 (219) | 43.1 (257) | 9.2 (55) | 13.5 (81) |
| ***Thailand*** | | | | | | | | |
| 12-14 | 493 | 8 [8-9] | 19 [18-21] | 0 [0-5] | 14.6 (67) | 22.1 (102) | 6.5 (30) | 7.7 (35) |
| 15-17 | 474 | 8 [8-9] | 20 [18-22] | 4 [0-6] | 13.7 (69) | 16.6 (84) | 4.4 (22) | 7.3 (37) |
| ***Uganda*** | | | | | | | | |
| 12-14 | 201 | 7 [5-9] | 18 [16-20] | 3 [0-5] | 38.8 (115) | 46.5 (138) | 35.3 (105) | 44.5 (132) |
| 15-17 | 815 | 6 [5-8] | 18 [16-21] | 3 [1-5] | 43.3 (311) | 49.9 (359) | 30.6 (220) | 56.1 (403) |
| ***Vietnam*** | | | | | | | | |
| 12-14 | 576 | 9 [8-9] | 19 [18-20] | 0 [0-1] | 29.4 (142) | 17.6 (85) | 2.7 (13) | 6.7 (33) |
| 15-17 | 418 | 9 [8-9] | 19 [18-21] | 0 [0-1] | 40.1 (204) | 18.9 (97) | 4.3 (22) | 7.2 (36) |

Mdn: median, IQR: interquartile range [Q1 first quartile - Q3 third quartile]. * Missing data for wellbeing items in Mozambique were not imputed due to the proportion of missing data for the psychological wellbeing total score (43%). ^a^Non-imputed n.

**Table S8. Mental health and wellbeing estimates by age and country with non-imputed data.**

| **Age** | **n** | **Life satisfaction**  Mdn [Q1-Q3] | **Psychological wellbeing**  Mdn [Q1-Q3] | **Anxiety**  Mdn [Q1-Q3] | **Depression**  %(n) | **Loneliness**  %(n) | **Self-harm**  %(n) | **Suicidal ideation/ attempts**  %(n) |
| --- | --- | --- | --- | --- | --- | --- | --- | --- |
| ***Cambodia*** | | | | | | | | |
| 12-14 | 535 | 6 [5-8] | 18 [16-19] | 2 [0-4] | 32.8 (142) | 35.6 (151) | 7.2 (32) | 17.1 (67) |
| 15-17 | 457 | 6 [5-8] | 18 [16-19] | 2 [0-5] | 42.5 (191) | 46.8 (213) | 8.2 (40) | 18.7 (80) |
| ***Ethiopia*** | | | | | | | | |
| 12-14 | 185 | 7 [5-10] | 19 [17-21] | 0 [0-2] | 36.3 (86) | 24.2 (56) | 5.4 (11) | 26 (56) |
| 15-17 | 815 | 6 [5-8] | 18 [17-21] | 2 [0-4] | 52 (358) | 39.6 (272) | 12 (75) | 32.2 (192) |
| ***Indonesia*** | | | | | | | | |
| 12-14 | 496 | 8 [7-9] | 18 [17-19] | 0 [0-3] | 6.7 (27) | 17.7 (71) | 4.3 (19) | 8.6 (38) |
| 15-17 | 499 | 8 [7-9] | 18 [17-19] | 1 [0-4] | 14.2 (65) | 26.6 (120) | 2.2 (11) | 8.3 (41) |
| ***Kenya*** | | | | | | | | |
| 12-14 | 420 | 7 [6-9] | 19 [17-21] | 2 [0-4] | 29.7 (121) | 36.1 (153) | 25.9 (109) | 36.2 (153) |
| 15-17 | 594 | 7 [5-8] | 20 [18-22] | 2 [0-5] | 30.7 (149) | 41.1 (204) | 26.5 (140) | 44.7 (228) |
| ***Malaysia*** | | | | | | | | |
| 12-14 | 535 | 8 [7-9] | 18 [17-21] | 0 [0-4] | 19.3 (84) | 19.6 (83) | 2.1 (10) | 5.5 (24) |
| 15-17 | 460 | 8 [7-9] | 18 [17-21] | 1 [0-4] | 30.2 (144) | 30.9 (148) | 2.9 (14) | 8.1 (40) |
| ***Mozambique*** | | | | | | | | |
| 12-14 | 262 | 6 [5-8] | 18 [15-19] | 3 [0-6] | 21.9 (66) | 31.7 (103) | 12.1 (40) | 20.8 (68) |
| 15-17 | 737 | 6 [5-8] | 18 [15-19] | 4 [1-7] | 26.6 (155) | 37.2 (223) | 10.4 (64) | 31.5 (196) |
| ***Namibia*** | | | | | | | | |
| 12-14 | 300 | 8 [6-9] | 19 [18-23] | 1 [0-5] | 21.6 (83) | 35.4 (136) | 8.7 (36) | 10.6 (45) |
| 15-17 | 694 | 7 [6-9] | 19 [18-22] | 3 [0-5] | 27 (127) | 47.6 (226) | 14.7 (78) | 20.4 (109) |
| ***Philippines*** | | | | | | | | |
| 12-14 | 489 | 7 [5-9] | 18 [16-19] | 5 [2-7] | 31.9 (120) | 67.4 (259) | 6.8 (29) | 26.8 (100) |
| 15-17 | 461 | 7 [5-8] | 17 [16-19] | 5 [3-7] | 39.8 (148) | 76.6 (298) | 10.9 (49) | 33.7 (131) |
| ***Tanzania*** | | | | | | | | |
| 12-14 | 319 | 5 [5-7] | 18 [16-21] | 0 [0-4] | 29.2 (107) | 35.6 (132) | 10.8 (43) | 8 (31) |
| 15-17 | 677 | 5 [4-7] | 19 [17-22] | 0 [0-4] | 36.5 (201) | 43.2 (238) | 9.1 (52) | 13.8 (78) |
| ***Thailand*** | | | | | | | | |
| 12-14 | 493 | 8 [8-9] | 20 [18-21] | 0 [0-5] | 14.2 (64) | 21.7 (98) | 6.1 (27) | 7.5 (33) |
| 15-17 | 474 | 8 [8-9] | 20 [18-22] | 4 [0-6] | 13 (65) | 16.2 (82) | 3.7 (18) | 7 (35) |
| ***Uganda*** | | | | | | | | |
| 12-14 | 201 | 7 [5-9] | 18 [16-20] | 3 [0-5] | 38.6 (105) | 46 (127) | 35.5 (101) | 45.9 (127) |
| 15-17 | 815 | 6 [5-8] | 18 [16-21] | 3 [1-5] | 42.4 (258) | 49.6 (325) | 30 (202) | 57.8 (389) |
| ***Vietnam*** | | | | | | | | |
| 12-14 | 576 | 9 [8-9] | 19 [18-20] | 0 [0-1] | 28.9 (132) | 16.5 (74) | 2.5 (12) | 6.4 (30) |
| 15-17 | 418 | 9 [8-9] | 19 [18-21] | 0 [0-1] | 40.2 (191) | 17.7 (83) | 4 (20) | 6.3 (31) |

Mdn: median, IQR: interquartile range [Q1 first quartile - Q3 third quartile]. Low counts of 5-9 have been rounded up to 10.

**Table S9. Multivariable regression analyses of the associations between age, gender, and food insecurity and mental health and wellbeing estimates using imputed data.**

| **Country** | **Age** | | **Gender** | | **Gender:Age** | | **Food security** | | | |
| --- | --- | --- | --- | --- | --- | --- | --- | --- | --- | --- |
|  | Estimate | p-value | Estimate | p-value | Estimate | p-value | Estimate (linear) | Estimate (quadratic) | Estimate (cubic) | Wald test  p-value |
| ***Life satisfaction*** | | | | | | | | | | |
| Cambodia | -0.06 | 0.348 | -0.07 | 0.961 | 0.00 | 0.969 | -0.15 | 0.59 | -0.32 | 0.213 |
| Ethiopia | -0.37 | **< 0.001*** | -2.79 | 0.175 | 0.18 | 0.168 | 1.03^a^ | 0.00 | -0.20 | **< 0.001*** |
| Indonesia | -0.03 | 0.544 | -0.53 | 0.607 | 0.03 | 0.683 | 0.09 | -0.24 | 0.05 | 0.636 |
| Kenya | -0.14 | **0.014*** | 1.17 | 0.347 | -0.08 | 0.316 | 0.41 | -0.15 | 0.29 | 0.308 |
| Malaysia | -0.10 | **0.035*** | 0.24 | 0.808 | -0.03 | 0.632 | 0.03 | 0.57 | -0.27 | 0.074 |
| Mozambique | -0.12 | 0.109 | -1.03 | 0.569 | 0.08 | 0.495 | 0.72^a^ | 0.15 | 0.09 | **0.005*** |
| Namibia | -0.05 | 0.369 | -0.64 | 0.65 | 0.04 | 0.627 | 0.18 | -0.47^a^ | 0.84^a^ | **< 0.001*** |
| Philippines | -0.15 | **0.048*** | -2.81 | **0.045*** | 0.19 | **0.048*** | 0.40 | 0.09 | -0.25 | 0.335 |
| Tanzania | -0.07 | 0.278 | -0.67 | 0.634 | 0.06 | 0.522 | 0.75^a^ | 0.94^a^ | -0.25 | **< 0.001*** |
| Thailand | 0.04 | 0.345 | 1.02 | 0.211 | -0.06 | 0.255 | 0.24 | 0.21 | -0.01 | **0.032*** |
| Uganda | -0.19 | **0.007*** | -2.56 | 0.181 | 0.18 | 0.147 | 0.65 | 0.37 | -0.49^a^ | **< 0.001*** |
| Vietnam | 0.02 | 0.63 | 0.72 | 0.274 | -0.05 | 0.3 | 0.40 | 0.40 | -0.16 | **< 0.001*** |
| ***Psychological wellbeing*** | | | | | | | | | | |
| Cambodia | 0.07 | 0.4 | 0.00 | 0.998 | 0.01 | 0.938 | -0.03 | 1.18^a^ | -0.52 | **0.012*** |
| Ethiopia | -0.04 | 0.744 | 3.68 | 0.15 | -0.21 | 0.187 | 1.08^a^ | 0.08 | -0.10 | **0.003*** |
| Indonesia | 0.24 | **0.013*** | 1.89 | 0.31 | -0.09 | 0.466 | 1.12 | -0.56 | 0.34 | 0.176 |
| Kenya | 0.36 | **< 0.001*** | 1.83 | 0.343 | -0.12 | 0.335 | 1.84^a^ | -0.33 | -0.34 | **< 0.001*** |
| Malaysia | -0.07 | 0.442 | -0.11 | 0.955 | -0.03 | 0.826 | 0.40 | 1.18^a^ | -0.91 | **0.021*** |
| Namibia | 0.18 | 0.088 | 3.77 | 0.113 | -0.22 | 0.15 | -0.19 | 0.41 | -0.01 | 0.65 |
| Philippines | -0.26 | **0.019*** | -4.93 | **0.019*** | 0.34 | **0.018*** | 0.96 | -0.68 | 0.17 | 0.799 |
| Tanzania | 0.28 | **0.001*** | 0.75 | 0.713 | -0.03 | 0.795 | 1.33^a^ | -0.29 | -0.95^a^ | **0.004*** |
| Thailand | 0.26 | **0.02*** | 0.82 | 0.67 | -0.03 | 0.84 | 0.95 | 0.88 | -1.18 | 0.202 |
| Uganda | 0.18 | 0.062 | -1.60 | 0.513 | 0.11 | 0.488 | 0.68 | 1.09^a^ | -0.11 | **< 0.001*** |
| Vietnam | 0.07 | 0.405 | 1.43 | 0.399 | -0.09 | 0.469 | 0.40 | 0.49 | 0.77 | **< 0.001*** |
| ***Anxiety*** | | | | | | | | | | |
| Cambodia | 0.17 | **0.046*** | 0.93 | 0.574 | -0.05 | 0.688 | 0.15 | -0.51 | 0.07 | 0.547 |
| Ethiopia | 0.24 | **0.017*** | -2.66 | 0.199 | 0.17 | 0.187 | -0.51 | -0.16 | 0.41 | **0.047*** |
| Indonesia | 0.15 | 0.065 | -1.91 | 0.251 | 0.16 | 0.174 | -0.46 | 0.12 | -0.16 | 0.598 |
| Kenya | 0.09 | 0.314 | -1.72 | 0.315 | 0.12 | 0.31 | 0.19 | -0.15 | 0.23 | 0.846 |
| Malaysia | -0.01 | 0.883 | -1.57 | 0.376 | 0.18 | 0.158 | -0.68 | -1.08^a^ | 0.04 | **< 0.001*** |
| Mozambique | 0.18 | 0.077 | -0.90 | 0.676 | 0.08 | 0.575 | 0.85^a^ | -0.33 | 0.50^a^ | **0.014*** |
| Namibia | 0.19 | 0.057 | -0.11 | 0.957 | -0.01 | 0.959 | 0.04 | -0.23 | -0.27 | 0.546 |
| Philippines | -0.03 | 0.742 | -2.61 | 0.155 | 0.21 | 0.095 | 1.17^a^ | -0.70 | 0.84^a^ | **0.005*** |
| Tanzania | -0.05 | 0.473 | -2.94 | 0.089 | 0.20 | 0.089 | 0.23 | -0.77 | 0.28 | 0.06 |
| Thailand | 0.11 | 0.322 | -3.26 | 0.106 | 0.23 | 0.107 | 0.74^a^ | -2.43^a^ | 0.12 | **< 0.001*** |
| Uganda | 0.02 | 0.782 | -1.26 | 0.544 | 0.10 | 0.432 | 0.47 | -0.55 | 0.05 | 0.28 |
| Vietnam | -0.03 | 0.517 | 0.67 | 0.574 | -0.04 | 0.637 | -0.47 | -1.51^a^ | 1.41 | **0.042*** |
| ***Depression*** | | | | | | | | | | |
| Cambodia | 0.12 | 0.051 | -0.86 | 0.505 | 0.04 | 0.662 | -0.08 | -0.37 | -0.06 | 0.245 |
| Ethiopia | 0.13 | 0.073 | -3.14 | 0.086 | 0.20 | 0.076 | -0.66 | 0.19 | 0.17 | 0.054 |
| Indonesia | 0.30 | **0.008*** | 1.59 | 0.472 | -0.09 | 0.538 | 0.17 | -0.80 | 0.25 | 0.157 |
| Kenya | -0.01 | 0.923 | -1.05 | 0.427 | 0.07 | 0.421 | -0.67^a^ | 0.50^a^ | 0.26 | **0.027*** |
| Malaysia | 0.24 | **0.004*** | 2.75 | 0.082 | -0.15 | 0.166 | -0.48 | -0.80^a^ | -0.02 | **< 0.001*** |
| Mozambique | 0.00 | 0.997 | -3.03 | 0.079 | 0.20 | 0.068 | 0.16 | -0.05 | 0.29 | 0.393 |
| Namibia | 0.07 | 0.371 | -1.52 | 0.397 | 0.10 | 0.386 | -0.07 | 0.34 | -0.42 | 0.295 |
| Philippines | 0.00 | 0.981 | -2.32 | 0.109 | 0.17 | 0.081 | -0.04 | -0.30 | 0.35 | 0.466 |
| Tanzania | 0.07 | 0.22 | -0.91 | 0.537 | 0.07 | 0.451 | 0.61^a^ | -1.18^a^ | 0.26 | **< 0.001*** |
| Thailand | -0.09 | 0.324 | -1.05 | 0.551 | 0.09 | 0.463 | -1.07^a^ | -0.72^a^ | 1.06^a^ | **< 0.001*** |
| Uganda | 0.20 | **0.008*** | 2.34 | 0.163 | -0.13 | 0.226 | 0.10 | -0.04 | 0.02 | 0.977 |
| Vietnam | 0.20 | **0.005*** | 0.64 | 0.665 | -0.04 | 0.703 | -0.59 | -0.05 | -0.13 | 0.122 |
| ***Loneliness*** | | | | | | | | | | |
| Cambodia | 0.09 | 0.147 | -1.06 | 0.402 | 0.05 | 0.561 | 0.32 | -0.69 | 0.14 | 0.081 |
| Ethiopia | 0.26 | **0.002*** | 0.19 | 0.927 | 0.00 | 0.979 | -0.02 | -0.33 | 0.29 | 0.133 |
| Indonesia | 0.11 | 0.183 | 0.31 | 0.842 | -0.01 | 0.961 | -0.31 | -0.09 | -0.03 | 0.439 |
| Kenya | 0.04 | 0.495 | -0.50 | 0.691 | 0.04 | 0.659 | -0.21 | 0.01 | 0.25 | 0.406 |
| Malaysia | 0.24 | **0.002*** | 3.30 | **0.03*** | -0.20 | 0.054 | -0.11 | -1.08^a^ | 0.14 | **< 0.001*** |
| Mozambique | 0.10 | 0.13 | -1.68 | 0.292 | 0.11 | 0.28 | 0.20 | 0.16 | 0.23 | 0.239 |
| Namibia | 0.21 | **0.003*** | 0.83 | 0.581 | -0.05 | 0.612 | 0.14 | 0.11 | 0.07 | 0.636 |
| Philippines | 0.07 | 0.378 | -1.27 | 0.405 | 0.11 | 0.294 | -0.20 | -0.06 | 0.29 | 0.623 |
| Tanzania | 0.07 | 0.227 | -1.31 | 0.365 | 0.10 | 0.308 | 0.67^a^ | -1.00^a^ | 0.40 | **< 0.001*** |
| Thailand | -0.05 | 0.582 | 1.61 | 0.311 | -0.12 | 0.273 | -1.27^a^ | -0.21 | 0.54 | **< 0.001*** |
| Uganda | 0.10 | 0.116 | 0.82 | 0.598 | -0.04 | 0.67 | 0.05 | -0.08 | 0.12 | 0.932 |
| Vietnam | 0.07 | 0.375 | 0.77 | 0.669 | -0.06 | 0.623 | -0.50 | -0.90 | 0.46 | **0.014*** |
| ***Selfharm*** | | | | | | | | | | |
| Cambodia | -0.02 | 0.854 | 0.78 | 0.727 | -0.05 | 0.729 | 0.31 | -1.01 | 0.37 | 0.092 |
| Ethiopia | 0.19 | 0.182 | -0.79 | 0.81 | 0.07 | 0.716 | -0.70 | 0.41 | -0.45 | 0.205 |
| Indonesia | -0.22 | 0.191 | -0.47 | 0.89 | 0.04 | 0.861 | -0.35 | -0.68 | 0.35 | 0.407 |
| Kenya | 0.02 | 0.706 | -0.16 | 0.908 | 0.00 | 0.962 | -0.07 | 0.01 | 0.20 | 0.821 |
| Malaysia | -0.02 | 0.902 | -2.48 | 0.508 | 0.17 | 0.518 | 6.28 | -5.59 | 3.40 | 0.114 |
| Mozambique | 0.05 | 0.663 | -1.35 | 0.604 | 0.07 | 0.697 | 0.45 | -0.29 | 0.21 | 0.573 |
| Namibia | 0.16 | 0.064 | -2.20 | 0.294 | 0.14 | 0.291 | 0.02 | 0.16 | 0.22 | 0.708 |
| Philippines | 0.13 | 0.333 | 0.85 | 0.715 | -0.05 | 0.773 | -1.02 | 0.47 | 0.25 | 0.092 |
| Tanzania | -0.02 | 0.806 | 0.44 | 0.851 | -0.02 | 0.911 | 0.02 | -0.27 | 0.24 | 0.817 |
| Thailand | -0.08 | 0.623 | 2.32 | 0.391 | -0.13 | 0.482 | -0.16 | -1.00 | 0.30 | 0.093 |
| Uganda | -0.07 | 0.296 | -1.28 | 0.434 | 0.09 | 0.411 | -0.28 | 0.12 | 0.00 | 0.675 |
| Vietnam | 0.16 | 0.361 | -0.86 | 0.827 | 0.06 | 0.82 | -0.81 | -0.79 | 0.87 | 0.21 |
| ***Suicidal ideation/attempts*** | | | | | | | | | | |
| Cambodia | -0.06 | 0.436 | -1.20 | 0.48 | 0.07 | 0.535 | 0.15 | -0.30 | -0.17 | 0.618 |
| Ethiopia | 0.20 | **0.023*** | 2.24 | 0.272 | -0.12 | 0.342 | -0.19 | 0.16 | -0.02 | 0.956 |
| Indonesia | -0.27 | **0.025*** | -5.11 | **0.02*** | 0.38 | **0.012*** | 0.30 | -0.28 | -0.06 | 0.874 |
| Kenya | 0.03 | 0.66 | -2.20 | 0.079 | 0.17 | **0.046*** | -0.48 | 0.48 | 0.09 | 0.126 |
| Malaysia | 0.14 | 0.343 | 2.74 | 0.294 | -0.14 | 0.416 | -0.45 | -1.09^a^ | 0.72 | **0.007*** |
| Mozambique | 0.18 | **0.018*** | 0.37 | 0.832 | -0.02 | 0.845 | -0.12 | 0.04 | 0.16 | 0.641 |
| Namibia | 0.16 | 0.065 | -2.32 | 0.235 | 0.16 | 0.203 | -0.10 | -0.13 | 0.08 | 0.831 |
| Philippines | -0.03 | 0.766 | -1.97 | 0.222 | 0.15 | 0.17 | -0.27 | -0.28 | 0.49 | 0.09 |
| Tanzania | 0.21 | **0.032*** | 2.12 | 0.318 | -0.10 | 0.46 | 0.86 | -0.25 | 0.12 | 0.277 |
| Thailand | 0.05 | 0.753 | 2.17 | 0.402 | -0.11 | 0.522 | -0.71 | -0.55 | 0.25 | 0.052 |
| Uganda | 0.15 | **0.028*** | -0.49 | 0.762 | 0.05 | 0.593 | -0.22 | 0.39 | 0.12 | 0.242 |
| Vietnam | 0.03 | 0.832 | -0.90 | 0.724 | 0.08 | 0.643 | 0.37 | -1.69^a^ | 0.68 | **0.009*** |

*Significant at <.05; ^a^Individual components (linear, quadratic, cubic) significant at <.05; gender was coded as 0 for male and 1 for female.

**Table S10. Regression analyses of age, gender and food insecurity on mental health and wellbeing estimates using non-imputed data.**

| **Country** | **Age** | | **Gender** | | **Gender:age** | | **Food security** | | | |
| --- | --- | --- | --- | --- | --- | --- | --- | --- | --- | --- |
|  | Estimate | p-value | Estimate | p-value | Estimate | p-value | Estimate factor (linear) | Estimate factor (quadratic) | Estimate factor (cubic) | p-value^a^ |
| ***Life satisfaction*** | | | | | | | | | | |
| Cambodia | -0.05 | 0.4 | -0.01 | 0.995 | -0.01 | 0.92 | -0.99 | -0.61 | -0.36 | 0.193 |
| Ethiopia | -0.35 | **< 0.001*** | -2.62 | 0.207 | 0.17 | 0.199 | 0.23 | 1.01 | 1.29 | **< 0.001*** |
| Indonesia | -0.03 | 0.562 | -0.79 | 0.417 | 0.05 | 0.47 | 0.36 | 0.31 | 0.16 | 0.558 |
| Kenya | -0.15 | **0.008*** | 0.94 | 0.45 | -0.07 | 0.42 | 0.60 | 0.39 | 0.68 | 0.304 |
| Malaysia | -0.09 | 0.06 | 0.37 | 0.709 | -0.04 | 0.538 | -0.75 | -0.45 | -0.09 | 0.073 |
| Mozambique | -0.15 | 0.052 | -1.73 | 0.365 | 0.13 | 0.291 | 0.26 | 0.50 | 1.07 | **0.004*** |
| Namibia | -0.07 | 0.225 | -0.96 | 0.496 | 0.07 | 0.476 | 1.34 | 0.27 | 0.64 | **< 0.001*** |
| Philippines | -0.13 | 0.09 | -2.55 | 0.077 | 0.17 | 0.083 | -0.14 | 0.37 | 0.43 | 0.331 |
| Tanzania | -0.07 | 0.282 | -0.66 | 0.64 | 0.06 | 0.519 | -0.84 | -0.16 | 0.88 | **< 0.001*** |
| Thailand | 0.04 | 0.448 | 0.93 | 0.256 | -0.06 | 0.298 | -0.13 | 0.00 | 0.30 | **0.038*** |
| Uganda | -0.20 | **0.007*** | -2.24 | 0.252 | 0.16 | 0.208 | -0.54 | 0.42 | 0.64 | **< 0.001*** |
| Vietnam | 0.02 | 0.586 | 0.79 | 0.231 | -0.05 | 0.257 | -0.37 | 0.03 | 0.46 | **< 0.001*** |
| ***Psychological wellbeing*** | | | | | | | | | | |
| Cambodia | 0.07 | 0.383 | 0.30 | 0.862 | 0.00 | 0.993 | -1.69 | -0.98 | -0.23 | **0.012*** |
| Ethiopia | -0.07 | 0.575 | 3.07 | 0.221 | -0.17 | 0.295 | 0.66 | 1.48 | 1.80 | **< 0.001*** |
| Indonesia | 0.23 | **0.025*** | 1.95 | 0.316 | -0.10 | 0.449 | 1.25 | 1.33 | 1.60 | 0.212 |
| Kenya | 0.41 | **< 0.001*** | 1.80 | 0.359 | -0.13 | 0.329 | 0.98 | 2.30 | 2.40 | **< 0.001*** |
| Malaysia | -0.13 | 0.192 | -2.24 | 0.253 | 0.11 | 0.404 | -1.43 | -0.25 | 0.30 | **0.014*** |
| Mozambique | 0.08 | 0.589 | -4.36 | 0.225 | 0.28 | 0.243 | 0.36 | 0.50 | 0.94 | 0.602 |
| Namibia | 0.15 | 0.176 | 3.76 | 0.134 | -0.22 | 0.166 | -0.52 | -0.76 | -0.17 | 0.228 |
| Philippines | -0.23 | 0.067 | -4.32 | 0.061 | 0.30 | 0.055 | 1.24 | 1.29 | 1.27 | 0.915 |
| Tanzania | 0.28 | **0.001*** | 0.82 | 0.69 | -0.03 | 0.804 | -0.13 | 1.72 | 1.23 | **0.007*** |
| Thailand | 0.23 | **0.048*** | 1.36 | 0.487 | -0.06 | 0.658 | -2.15 | 0.04 | 0.22 | 0.463 |
| Uganda | 0.16 | 0.113 | -1.83 | 0.47 | 0.12 | 0.448 | -1.22 | -0.68 | 0.65 | **< 0.001*** |
| Vietnam | 0.09 | 0.305 | 1.21 | 0.477 | -0.06 | 0.59 | 0.87 | -0.30 | 0.95 | **< 0.001*** |
| ***Anxiety*** | | | | | | | | | | |
| Cambodia | 0.16 | 0.064 | 0.99 | 0.558 | -0.05 | 0.681 | 0.77 | 0.74 | 0.34 | 0.433 |
| Ethiopia | 0.23 | **0.027*** | -3.16 | 0.134 | 0.20 | 0.126 | 0.22 | -0.55 | -0.59 | **0.046*** |
| Indonesia | 0.18 | **0.029*** | -1.03 | 0.54 | 0.09 | 0.427 | -0.57 | -0.58 | -0.79 | 0.531 |
| Kenya | 0.13 | 0.118 | -0.65 | 0.697 | 0.04 | 0.702 | 0.43 | 0.20 | 0.42 | 0.761 |
| Malaysia | 0.00 | 0.951 | -1.49 | 0.401 | 0.17 | 0.164 | 0.66 | 0.84 | -0.56 | **< 0.001*** |
| Mozambique | 0.20 | 0.065 | -1.14 | 0.631 | 0.11 | 0.481 | 1.23 | 0.70 | 1.42 | **0.007*** |
| Namibia | 0.19 | 0.061 | -0.44 | 0.836 | 0.02 | 0.89 | 0.14 | 0.42 | -0.05 | 0.537 |
| Philippines | -0.11 | 0.306 | -3.47 | 0.074 | 0.27 | **0.039*** | 2.00 | 1.36 | 1.87 | **0.015*** |
| Tanzania | -0.03 | 0.635 | -2.56 | 0.144 | 0.17 | 0.142 | 1.14 | 0.87 | 0.45 | 0.061 |
| Thailand | 0.11 | 0.322 | -3.49 | 0.088 | 0.24 | 0.086 | 2.68 | 3.28 | 1.23 | **< 0.001*** |
| Uganda | 0.03 | 0.761 | -1.16 | 0.594 | 0.10 | 0.467 | 0.72 | 1.01 | 0.63 | 0.269 |
| Vietnam | -0.03 | 0.515 | 0.78 | 0.502 | -0.05 | 0.551 | 2.82 | 0.37 | 0.01 | 0.104 |
| ***Depression*** | | | | | | | | | | |
| Cambodia | 0.09 | 0.148 | -1.16 | 0.38 | 0.06 | 0.508 | 0.37 | 0.32 | -0.15 | 0.198 |
| Ethiopia | 0.14 | 0.06 | -2.78 | 0.136 | 0.18 | 0.13 | -0.49 | -1.05 | -0.94 | **0.035*** |
| Indonesia | 0.31 | **0.008*** | 1.75 | 0.456 | -0.09 | 0.543 | 1.17 | 0.94 | 0.34 | 0.089 |
| Kenya | -0.01 | 0.866 | -0.96 | 0.472 | 0.06 | 0.48 | -0.46 | -1.26 | -0.82 | **0.011*** |
| Malaysia | 0.23 | **0.008*** | 2.42 | 0.141 | -0.13 | 0.264 | 0.70 | 0.45 | -0.66 | **< 0.001*** |
| Mozambique | 0.00 | 0.96 | -3.70 | **0.036*** | 0.25 | **0.03*** | 0.38 | -0.06 | 0.29 | 0.228 |
| Namibia | 0.08 | 0.372 | -1.31 | 0.504 | 0.09 | 0.465 | -0.81 | -0.23 | -0.31 | 0.212 |
| Philippines | 0.00 | 0.97 | -2.46 | 0.117 | 0.19 | 0.083 | 0.62 | 0.09 | 0.05 | 0.367 |
| Tanzania | 0.06 | 0.333 | -1.08 | 0.474 | 0.08 | 0.395 | 1.95 | 1.80 | 1.04 | **< 0.001*** |
| Thailand | -0.13 | 0.158 | -1.70 | 0.341 | 0.13 | 0.275 | 1.02 | -0.71 | -0.95 | **< 0.001*** |
| Uganda | 0.21 | **0.005*** | 2.82 | 0.098 | -0.16 | 0.141 | 0.16 | 0.16 | 0.20 | 0.957 |
| Vietnam | 0.21 | **0.004*** | 0.72 | 0.623 | -0.04 | 0.662 | -0.14 | -0.28 | -0.79 | 0.058 |
| ***Loneliness*** | | | | | | | | | | |
| Cambodia | 0.08 | 0.184 | -1.21 | 0.353 | 0.06 | 0.511 | 1.02 | 0.96 | 0.54 | 0.073 |
| Ethiopia | 0.29 | **< 0.001*** | 0.53 | 0.798 | -0.02 | 0.899 | 0.59 | 0.18 | 0.13 | 0.144 |
| Indonesia | 0.10 | 0.219 | 0.24 | 0.879 | 0.00 | 0.987 | -0.14 | -0.22 | -0.55 | 0.267 |
| Kenya | 0.04 | 0.491 | -0.32 | 0.8 | 0.02 | 0.767 | 0.19 | -0.34 | -0.18 | 0.224 |
| Malaysia | 0.26 | **< 0.001*** | 3.49 | **0.025*** | -0.21 | **0.043*** | 1.60 | 1.25 | 0.25 | **< 0.001*** |
| Mozambique | 0.10 | 0.156 | -1.87 | 0.253 | 0.13 | 0.232 | 0.05 | -0.20 | 0.31 | 0.165 |
| Namibia | 0.25 | **< 0.001*** | 1.44 | 0.358 | -0.09 | 0.403 | 0.00 | -0.07 | 0.28 | 0.342 |
| Philippines | 0.04 | 0.612 | -1.91 | 0.239 | 0.16 | 0.162 | 0.19 | -0.33 | -0.22 | 0.59 |
| Tanzania | 0.07 | 0.265 | -1.24 | 0.397 | 0.09 | 0.334 | 1.78 | 1.44 | 1.11 | **< 0.001*** |
| Thailand | -0.07 | 0.401 | 1.32 | 0.408 | -0.10 | 0.362 | -0.03 | -1.14 | -1.47 | **< 0.001*** |
| Uganda | 0.09 | 0.158 | 0.29 | 0.855 | -0.01 | 0.934 | 0.29 | 0.11 | 0.15 | 0.836 |
| Vietnam | 0.08 | 0.329 | 0.99 | 0.579 | -0.08 | 0.543 | 0.63 | 0.29 | -0.46 | **0.018*** |
| ***Self-harm*** | | | | | | | | | | |
| Cambodia | -0.03 | 0.729 | 0.15 | 0.945 | 0.00 | 0.99 | 1.65 | 1.22 | 0.53 | **0.025*** |
| Ethiopia | 0.25 | **0.026*** | -1.05 | 0.73 | 0.10 | 0.611 | -1.45 | -1.05 | -1.48 | 0.061 |
| Indonesia | -0.25 | 0.147 | -1.06 | 0.77 | 0.08 | 0.741 | 0.85 | 0.10 | -0.42 | 0.359 |
| Kenya | 0.02 | 0.732 | -0.07 | 0.958 | 0.00 | 0.97 | 0.16 | -0.12 | 0.05 | 0.841 |
| Malaysia | 0.05 | 0.797 | -2.75 | 0.457 | 0.19 | 0.456 | 16.60 | 14.75 | 15.13 | **< 0.001*** |
| Mozambique | 0.02 | 0.86 | -1.55 | 0.566 | 0.08 | 0.642 | 0.93 | 0.79 | 0.87 | 0.355 |
| Namibia | 0.16 | 0.072 | -2.29 | 0.294 | 0.15 | 0.3 | -0.01 | -0.25 | 0.12 | 0.7 |
| Philippines | 0.16 | 0.277 | 1.34 | 0.598 | -0.08 | 0.647 | -0.71 | -1.65 | -1.39 | 0.051 |
| Tanzania | -0.03 | 0.736 | 0.37 | 0.875 | -0.01 | 0.934 | 0.50 | 0.18 | 0.12 | 0.793 |
| Thailand | -0.09 | 0.55 | 2.73 | 0.29 | -0.17 | 0.353 | 0.53 | 1.18 | 0.39 | 0.148 |
| Uganda | -0.08 | 0.236 | -1.02 | 0.535 | 0.07 | 0.499 | -0.23 | -0.38 | -0.42 | 0.574 |
| Vietnam | 0.17 | 0.34 | -1.28 | 0.751 | 0.08 | 0.762 | 0.51 | -0.48 | -0.74 | 0.617 |
| ***Suicidal ideation/attempts*** | | | | | | | | | | |
| Cambodia | -0.07 | 0.438 | -1.13 | 0.521 | 0.06 | 0.599 | 0.10 | 0.64 | 0.14 | 0.429 |
| Ethiopia | 0.22 | **0.019*** | 2.04 | 0.344 | -0.10 | 0.441 | -0.47 | -0.57 | -0.44 | 0.776 |
| Indonesia | -0.29 | **0.021*** | -5.03 | **0.025*** | 0.38 | **0.017*** | 0.42 | 0.59 | 0.41 | 0.88 |
| Kenya | 0.02 | 0.697 | -2.40 | 0.06 | 0.18 | **0.033*** | -0.58 | -0.96 | -0.52 | 0.093 |
| Malaysia | 0.18 | 0.239 | 3.31 | 0.229 | -0.18 | 0.328 | 1.97 | 0.70 | 0.05 | **0.005*** |
| Mozambique | 0.19 | **0.016*** | -0.10 | 0.958 | 0.01 | 0.9 | -0.06 | -0.38 | -0.19 | 0.427 |
| Namibia | 0.17 | 0.057 | -2.38 | 0.231 | 0.16 | 0.194 | 0.22 | 0.00 | -0.09 | 0.715 |
| Philippines | -0.02 | 0.824 | -1.82 | 0.283 | 0.14 | 0.232 | 1.00 | 0.02 | 0.07 | **0.024*** |
| Tanzania | 0.21 | **0.031*** | 2.08 | 0.326 | -0.10 | 0.47 | 0.88 | 1.06 | 1.30 | 0.263 |
| Thailand | 0.00 | 0.981 | 1.08 | 0.68 | -0.04 | 0.825 | 0.26 | 0.02 | -0.62 | 0.148 |
| Uganda | 0.16 | **0.016*** | -0.07 | 0.964 | 0.03 | 0.754 | -0.27 | -0.62 | -0.18 | 0.223 |
| Vietnam | 0.00 | 0.989 | -1.31 | 0.62 | 0.11 | 0.546 | 2.66 | 1.83 | 0.93 | **0.01*** |

^a^Wald test.

**Figure S1. Significant associations between mental health/wellbeing estimates and age and gender using imputed data.**

**
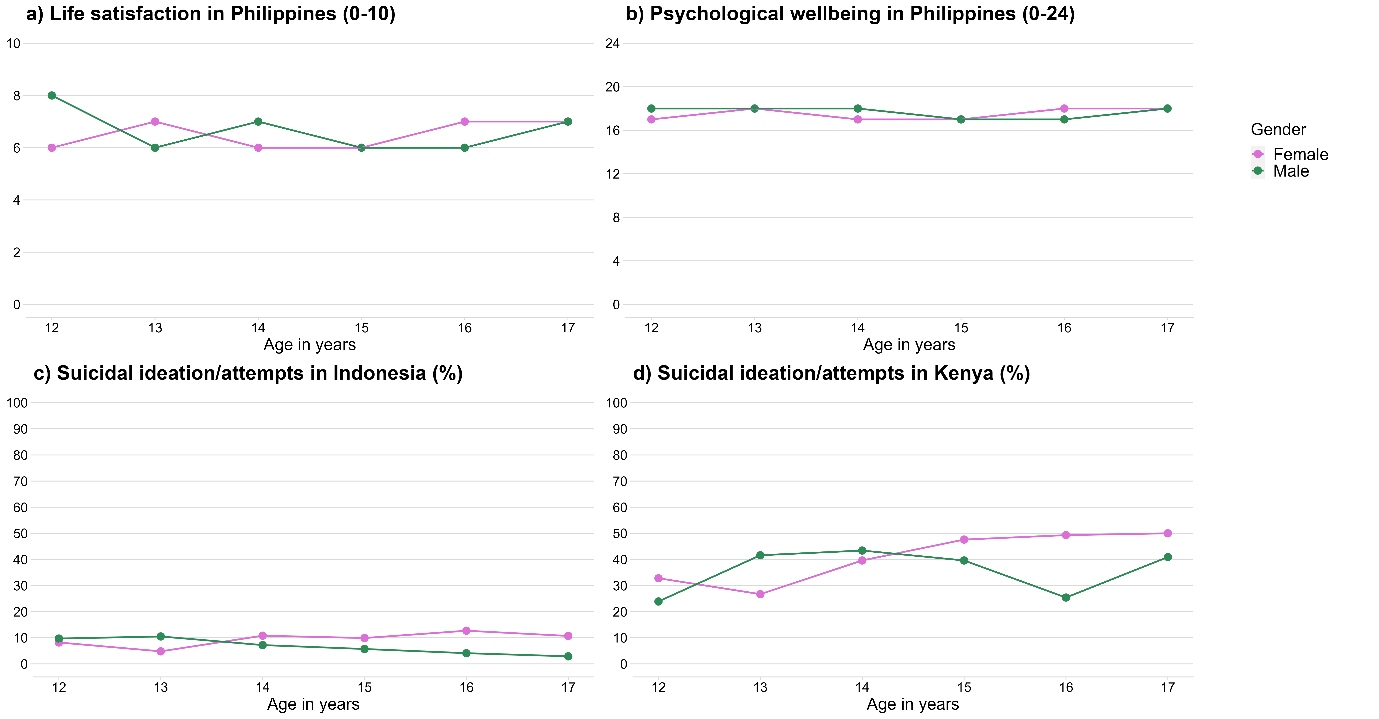
**
